# A c-di-GMP Effector System Controls Cell Adhesion by Inside-Out Signaling and Surface Protein Cleavage

**DOI:** 10.1371/journal.pbio.1000587

**Published:** 2011-02-01

**Authors:** Peter D. Newell, Chelsea D. Boyd, Holger Sondermann, George A. O'Toole

**Affiliations:** 1 Department of Microbiology and Immunology, Dartmouth Medical School, Hanover, New Hampshire, United States of America; 2 Department of Molecular Medicine, College of Veterinary Medicine, Cornell University, Ithaca, New York, United States of America; Massachusetts General Hospital/Harvard Medical School, United States of America

## Abstract

In *Pseudomonas fluorescens* Pf0-1 the availability of inorganic phosphate (Pi) is an environmental signal that controls biofilm formation through a cyclic dimeric GMP (c-di-GMP) signaling pathway. In low Pi conditions, a c-di-GMP phosphodiesterase (PDE) RapA is expressed, depleting cellular c-di-GMP and causing the loss of a critical outer-membrane adhesin LapA from the cell surface. This response involves an inner membrane protein LapD, which binds c-di-GMP in the cytoplasm and exerts a periplasmic output promoting LapA maintenance on the cell surface. Here we report how LapD differentially controls maintenance and release of LapA: c-di-GMP binding to LapD promotes interaction with and inhibition of the periplasmic protease LapG, which targets the N-terminus of LapA. We identify conserved amino acids in LapA required for cleavage by LapG. Mutating these residues in chromosomal *lapA* inhibits LapG activity in vivo, leading to retention of the adhesin on the cell surface. Mutations with defined effects on LapD's ability to control LapA localization in vivo show concomitant effects on c-di-GMP-dependent LapG inhibition in vitro. To establish the physiological importance of the LapD-LapG effector system, we track cell attachment and LapA protein localization during Pi starvation. Under this condition, the LapA adhesin is released from the surface of cells and biofilms detach from the substratum. This response requires c-di-GMP depletion by RapA, signaling through LapD, and proteolytic cleavage of LapA by LapG. These data, in combination with the companion study by Navarro et al. presenting a structural analysis of LapD's signaling mechanism, give a detailed description of a complete c-di-GMP control circuit—from environmental signal to molecular output. They describe a novel paradigm in bacterial signal transduction: regulation of a periplasmic enzyme by an inner membrane signaling protein that binds a cytoplasmic second messenger.

## Author Summary

Bacteria can live as free swimming cells or attached to surfaces in communities called biofilms. The di-nucleotide c-di-GMP is a key cytoplasmic signal that regulates biofilm formation in a number of bacterial species. Our study, in combination with structural analysis described in the accompanying paper by Sondermann et al., describes key interactions in a c-di-GMP signaling pathway that allows cells of *Pseudomonas fluorescens* to adapt to changes in the concentration of the nutrient phosphate by regulating biofilm formation. The adhesion protein LapA is localized outside the bacterial cell membrane and is responsible for keeping cells attached to surfaces. We show that under low phosphate conditions levels of c-di-GMP are depleted in cells, and these changes are sensed by LapD, a transmembrane c-di-GMP receptor protein. When c-di-GMP levels are low, the LapD protein is kept in an “off” state that allows LapG, a periplasmic protease, to interact with LapA and cleave the N-terminal domain of this adhesion, releasing LapA from the cell surface and promoting biofilm detachment. Under abundant phosphate conditions, LapD binds c-di-GMP in the cytoplasm and binds to and sequesters LapG in the periplasm, promoting cell adhesion via maintenance of LapA on the cell surface.

## Introduction

Bacteria can be exquisitely tuned to sense and respond to changes in their environment. A single cell may possess an immense repertoire of signal transduction systems capable of receiving sensory input and directing physiological adaptation. The recent groundswell of studies on the intracellular second messenger cyclic dimeric GMP (c-di-GMP) has added a new dimension to bacterial signaling. c-di-GMP controls major lifestyle transitions for bacteria, promoting the shift from motile to sessile modes of growth through impacts on diverse physiological outputs. This molecule is synthesized by diguanylate cyclases (DGCs) [1], proteins that contain the GGDEF domain, and can be degraded by specific phosphodiesterases (PDEs) containing either the EAL or HD-GYP domain [2],[3]. Such domains are ubiquitous in bacterial genomes, and occur in combination with an array of sensory input and output modules [4].

A substantial body of work has identified specific DGCs and PDEs that impact cell adhesion and biofilm formation in diverse bacteria. The phenotypic effects of these signaling proteins include changes in exopolysaccharide (EPS) production, motility, and transcription [5]. Assigning c-di-GMP signaling activity to many proteins, sometimes dozens within a single bacterium, has highlighted the complexities of c-di-GMP signaling networks, and has exacerbated the task of connecting specific environmental signals to discrete outputs.

A key, recent advance in our understanding c-di-GMP's role in bacteria has been the identification of c-di-GMP receptors with defined outputs. Receptors, or effector proteins, identified thus far utilize a range of c-di-GMP binding mechanisms to impact EPS synthesis [6],[7],[8], motility [9],[10],[11],[12], transcription [13],[14],[15], and sub-cellular [16] or cell-surface protein localization [17]. In a few cases, molecular details of the effector's output have been determined. c-di-GMP binding to the PilZ domain of YcgR stimulates its interaction with the flagellar complex of *E. coli*, resulting in a counter-clockwise rotational bias and reduced motility [18],[19],[20]. In *V. cholerae*, c-di-GMP binds the transcription factor VpsT causing a change in its oligomerization and activity, inversely regulating genes for rugosity and motility [15]. PopA of *C. crescentus* undergoes dynamic localization to the cell pole upon c-di-GMP binding, recruiting a cell cycle regulator for degradation [16]. In addition to binding effector proteins, c-di-GMP has also been shown to bind riboswitches [21],[22],[23]. The diversity of these control mechanisms, and their varied targets, highlights the scope and intricacy of c-di-GMP signaling. Despite the significant progress these studies represent, in most cases the environmental or cellular inputs controlling the DGCs and/or PDEs that regulate these effectors have yet to be defined.

Stable surface attachment and subsequent biofilm formation by *Pseudomonas fluorescens* Pf0-1 requires a large adhesive protein, LapA. This ∼520 kD protein is secreted to the surface of the outer membrane by an ABC transporter encoded by the *lapEBC* genes [24]. LapA's maintenance on the cell surface is controlled post-translationally by the c-di-GMP binding protein LapD [17]. When c-di-GMP levels are high, LapD binds c-di-GMP and promotes biofilm formation via accumulation of LapA on the cell surface. In the absence of c-di-GMP binding to LapD, LapA is released from the cell rendering it unable to attach [17].

In a prior study, our group characterized LapD, reporting genetic and biochemical evidence that LapD binds c-di-GMP through its cytoplasmic EAL domain and controls biofilm formation via a periplasmic output domain [17]. The structure/function analysis presented by Newell et al. suggested that LapD controls LapA localization by a unique inside-out signaling mechanism: binding c-di-GMP in the cytoplasm and transmitting this signal through the inner membrane to the periplasm via a HAMP domain. Such a mechanism could account for how changes in cytoplasmic c-di-GMP levels control LapA's stability on the cell surface post-translationally. However, the mechanism by which LapD's periplasmic domain impacted LapA localization was unknown.

The availability of inorganic phosphate (Pi) is an important environmental signal that governs biofilm formation by *P. fluorescens* Pf0-1 via a c-di-GMP-dependent mechanism. When Pi is limiting, the c-di-GMP PDE RapA is expressed and depletes cellular c-di-GMP, suppressing biofilm formation [25]. One effect of RapA's activity is the loss of the adhesin LapA from the cell surface. While our previous study showed that the effects of Pi starvation and RapA expression on biofilm require signaling through LapD [17], the specific contribution of LapD to changes in LapA localization in this signaling pathway was not known.

Here we uncover how LapD controls LapA localization and provide biochemical data describing its function as an inside-out signaling protein. When bound to c-di-GMP, LapD inhibits the activity of a periplasmic protease, LapG. In the absence of c-di-GMP binding to LapD, LapG is free to cleave the N-terminus of LapA, releasing the adhesin from the cell and preventing biofilm formation. Upon Pi starvation, the LapD-LapG system responds to c-di-GMP depletion by RapA and promotes biofilm detachment. These data, in combination with the companion study by Navarro et al. 26 presenting a structural analysis of LapD's signaling mechanism, describe a key connection in a complete c-di-GMP control circuit that links environmental signal to cellular output.

## Results

Note: The Supporting Information section includes an expanded alignment of LapA-like proteins (Figure S1), a graphical depiction of data describing inhibition of LapG activity by c-di-GMP and additional data on LapG activity in the presence of detergents (Figure S2), data describing the localization of control proteins in the presence and absence of c-di-GMP (Figure S3), and images of representative biofilm assays from the dataset depicted graphically in Figure 7B (Figure S4).

### Phenotypic and Genetic Analyses of the *lapG* Mutant

**Figure 1:**
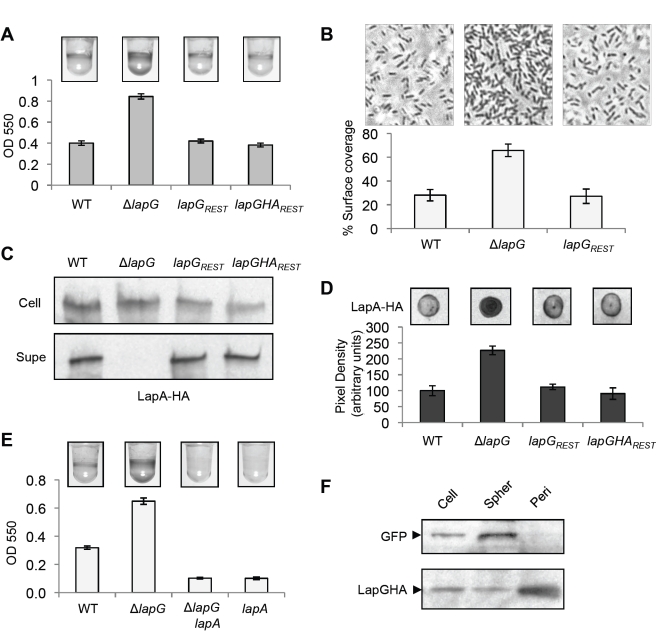
Phenotypic analyses of the *lapG* mutant. (A) A quantitative biofilm assay comparing the indicated strains. Crystal violet stained biomass (pictured above) was dissolved and quantified by spectrophotometry at 550 nm. Data for all biofilm assays are presented as the mean ± SD for 8–12 replicates. (B) Initial surface attachment was visualized by phase contrast microscopy, 1 h post-inoculation. Estimates of the percent surface coverage were calculated by densitometry on 8 independent fields of view for each strain (mean ± SD). The images shown are one-sixth of a field of view. (C) HA-tagged LapA protein from cell extracts (Cell) and culture supernatants (Supe) of the indicated strains is shown by Western blot, probed with anti-HA antibodies. (D) LapA on the surface of intact cells visualized by immunoblot (top), and quantified by densitometry (bottom; mean ± SD, *n* = 3). Pixel density values were scaled to set WT level at 100. (E) A biofilm assay examining the dependence of the Δ*lapG* mutant phenotype on *lapA*. (F) The abundance of LapGHA and GFP in whole cell (Cell), spheroplast (Spher), and periplasmic (Peri) fractions, after normalization to total protein concentration, is shown by Western blot.

In this study, our objective was to determine the mechanism by which the c-di-GMP effector LapD controls LapA localization. In an effort to identify additional players in this pathway, we investigated the function of a gene immediately upstream of *lapD*, designated *lapG*. We deleted the *lapG* gene (Pfl_0130) and determined the effects of this mutation on irreversible surface attachment and biofilm formation. After 6 h in a static culture, the *lapG* mutant (Δ*lapG*) showed a hyper-adherent biofilm phenotype, accumulating twice as much biomass on the culture well as the WT (Figure 1A). These strains were examined by microscopy under similar, static growth conditions. After a 1-h incubation, irreversibly attached Δ*lapG* cells covered twice as much of the substratum as compared to the WT (Figure 1B). Through longer incubation times, Δ*lapG* continued to show about twice as many attached cells as WT (unpublished data). These results suggest that increased cell attachment accounts for the biofilm phenotype of Δ*lapG*.

To complement the *lapG* mutant we reintroduced the gene on a multi-copy plasmid. This caused total loss of biofilm formation, shown and discussed in more detail below. A second approach was employed: restoring the *lapG* gene to its native locus in Δ*lapG* using allelic replacement. The resulting strain, *lapG_REST_*, showed similar levels of biofilm formation and surface attachment as WT (Figure 1A,B). When a *lapG* allele carrying an internal HA epitope tag (*lapG-HA*) was introduced into the *lapG* locus, this also restored the WT phenotype (Figure 1A).

The adhesin LapA is the primary factor required by *P. fluorescens* for attachment to surfaces under these conditions [17],[24],[25]. We hypothesized that increased expression or cell surface localization of LapA might account for the biofilm phenotype of Δ*lapG.* To test these hypotheses, we examined LapA levels in cell extracts and culture supernatants by Western blot, and on the surface of intact cells by dot blot. Cell extracts of WT and Δ*lapG* showed similar levels of LapA, suggesting comparable levels of LapA protein expression in these strains (Figure 1C; 0.98±0.05-fold change from WT, *n* = 3). Interestingly, the *lapG* mutant had a unique LapA localization phenotype: there was no detectable LapA in the supernatant and a 2-fold increase in LapA on the cell surface (Figure 1C,D). These data suggest that *lapG* is involved in the release of LapA from the cell surface. The Δ*lapG* phenotypes are consistent with previous data showing that cell-surface localization of LapA has a direct and proportional stimulatory effect on biofilm formation [17],[27]. Restoration of either the WT or *lapG-HA* alleles to the *lapG* locus of Δ*lapG* restored a WT LapA localization phenotype (Figure 1C,D).

If increased adhesion by Δ*lapG* is caused by the aberrant accumulation of LapA on the cell surface, then a mutation in *lapA* should be epistatic to *lapG*. Introduction of a null mutation in *lapA* into the Δ*lapG* mutant completely eliminated biofilm formation (Figure 1E), a phenotype identical to that of a *lapA* mutant.

### Subcellular Localization of LapG

LapG contains a putative Sec secretion signal (probability 0.85; www.cbs.dtu.dk/services/signalP) but no transmembrane domains, and thus is predicted to be periplasmic. To test this proposed localization of LapG, we used osmotic shock to release periplasmic proteins and compared the relative proportion of LapG in this periplasmic fraction versus the remaining spheroplasts. We utilized the *lapGHA_REST_* strain expressing GFP to provide a control for tracking cytoplasmic proteins. GFP localized exclusively to the spheroplasts, where it was enriched relative to whole cell lysates (Figure 1F). In contrast, LapGHA was enriched in the periplasmic fraction and depleted in the spheroplasts. These data suggest that LapG resides in the periplasm.

### LapG Modifies the LapA Protein

**Figure 2:**
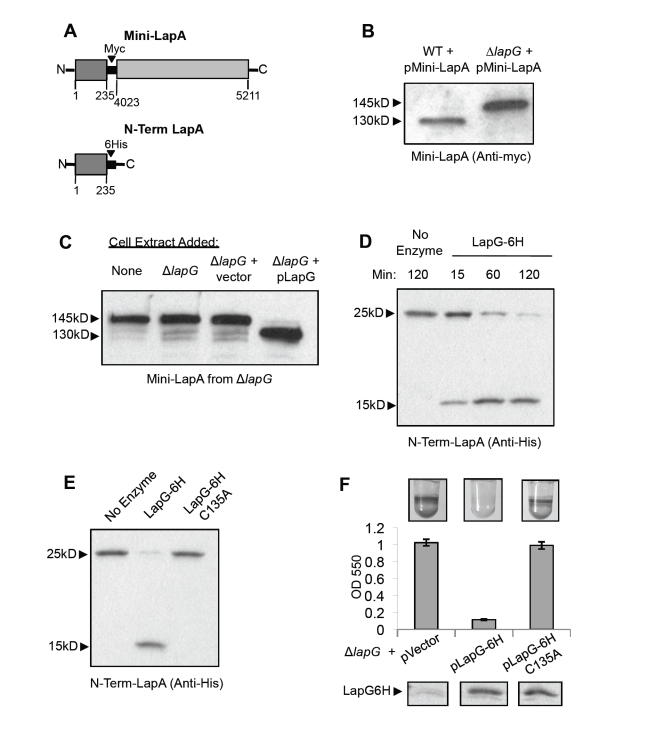
LapG modifies the LapA protein. (A) Diagrams of LapA constructs: Mini-LapA contains the N-terminal and C-terminal portions of LapA with 3,788 amino acids of the central, repetitive region of the protein replaced by a Myc tag. The predicted molecular weight of this protein is 145 kDa. N-term-LapA contains the N-terminal 235 amino acids of LapA fused to a 6H tag at its C-terminus. The predicted molecular weight of N-term LapA is 25 kDa. (B) Cell-associated fractions of WT and Δ*lapG* strains were probed for Mini-LapA by Western blot. The blot shown highlights the difference in apparent molecular weight of Mini-LapA from these strains. (C) Mini-LapA isolated from the Δ*lapG* strain was treated with cell extracts from the strains indicated above. Cleavage of Mini-LapA from 145 kDa to 130 kDa after treatment with a LapG-containing lysate is shown by Western blot. (D) Purified N-Term-LapA (25 kDa) is cleaved to 15 kDa in a time-dependent manner upon addition of purified LapG, visualized by Western blot. (E) Purified N-Term LapA is cleaved by LapG but not the LapG-C135A mutant, after 120 min of incubation in vitro, shown by Western blot. (F) A biofilm assay examining the ability of pLapG-6H C135A to reduce biofilm formation by the Δ*lapG* strain, relative with the empty vector (pMQ72) and pLapG-6H controls. *Below*: The abundance of LapG-6H and LapG-6H C135A in cell lysates is shown by Western blot.

Noting the absence of LapA in the supernatant and its accumulation on the cell surface of the *lapG* mutant, we hypothesized that LapG functions to modify and release LapA from the cell. A smaller variant of LapA, Mini-LapA, was generated to assess modification of this large protein (∼520 kDa). Mini-LapA consists of the N- and C-termini of LapA, flanking an internal myc-epitope tag (Figure 2A, top). Mini-LapA was introduced on a multi-copy plasmid to WT and Δ*lapG* strains and its secretion and cell-surface localization were assessed. Mini-LapA localized to the cell-associated (Figure 2B) and supernatant fractions (unpublished data), indicating that it is still secreted. However Mini-LapA does not appear to be functional for adhesion, as it cannot complement a *lapA* mutant (unpublished data). We observed a difference in the apparent size of secreted Mini-LapA isolated from strains with or without LapG: in the presence of LapG (i.e., the WT genetic background), Mini-LapA migrates at a smaller size, approximately 130 kDa. In contrast, Mini-LapA in Δ*lapG* migrates at its predicted size of 145 kDa (Figure 2B). This result suggests that LapG is required for a change in molecular weight of Mini-LapA. Given the predicted function of LapG, as a cysteine protease [28], the modification to Mini-LapA was likely proteolytic cleavage.

We developed an assay to assess the necessity of LapG for Mini-LapA modification. Unmodified Mini-LapA was prepared from a cell extract of the Δ*lapG* mutant overexpressing Mini-LapA. This Mini-LapA substrate was incubated at room temperature for 30 min with cell extracts prepared from the Δ*lapG* mutant, the Δ*lapG* mutant carrying an empty vector, and the Δ*lapG* mutant carrying a plasmid overexpressing LapG. Reactions were then analyzed by Western blotting to reveal that Mini-LapA modification only occurs in the presence of LapG (Figure 2C). This result suggests a model in which LapG functions to modify the LapA protein through proteolytic cleavage of 10–15 kDa from LapA.

### LapG Is a Cysteine Protease

We hypothesized that LapG cleaves 10–15 kDa from the N-terminus of Mini-LapA, as the C-terminus of LapA contains residues necessary for type I secretion. To test this hypothesis, we constructed another surrogate LapG substrate, N-Term-LapA, consisting of the first 235 amino acids of LapA with a 6xhistidine (6H) epitope tag at its C-terminus (Figure 2A, bottom). N-Term-LapA and LapG-6H were each purified by nickel affinity chromatography, then incubated together for 15, 60, and 120 min. Subsequent Western blotting revealed that LapG-6H is necessary and sufficient for N-Term-LapA modification in vitro (Figure 2D). Modification occurs in a time-dependent manner and results in a 10–15 kDa reduction in the apparent molecular weight of N-Term-LapA, consistent with the observations of Mini-LapA above.

LapG contains a conserved domain of unknown function (DUF920), proposed to constitute a family of Bacterial Transglutaminase-like Cysteine Proteinases (BTLCPs) [28]. In the study identifying BTLCPs, the authors note that BTLCPs contain a conserved C-H-D catalytic triad. We tested the requirement for the cysteine of LapG's catalytic triad for LapA modification, by mutating C135 of LapG to alanine. Even after incubation with N-Term-LapA for 2 h, purified LapG-C135A did not modify N-Term-LapA. As a control, the WT LapG completely converted the N-Term-LapA substrate in this time (Figure 2E).

Given the inactivity of LapG-C135A, we predicted that this mutation would disrupt LapG's function in vivo. We expressed this mutant on a multi-copy plasmid in the Δ*lapG* strain and assessed the effect of biofilm formation, relative to the WT allele. As mentioned above, expressing WT LapG from a plasmid resulted in a loss of biofilm formation (Figure 2F). The strain expressing the C135A mutant showed a hyper-adherent biofilm phenotype, comparable to that of the Δ*lapG* mutant. Together, these data show that LapG's cysteine residue is required for N-Term-LapA modification and that LapG's activity is required for WT biofilm formation. This suggests a model in which cleavage of the LapA protein by LapG is necessary for release of the adhesin from the cell.

### Appropriate Localization of LapA Requires a Functional LapG Cleavage Site

**Figure 3:**
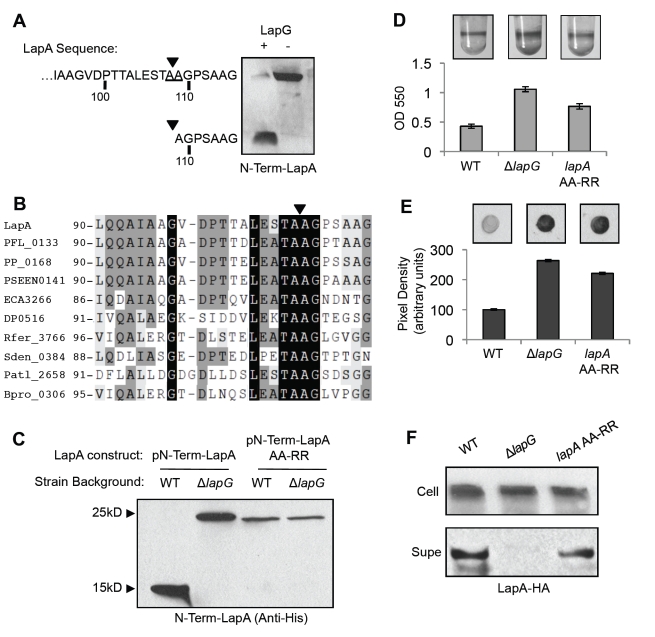
Functional analysis of LapA's cleavage site. (A) N-Term-LapA protein was sequenced by Edman degradation before and after treatment with LapG; the samples are shown analyzed by Western blot. The amino acid sequence determined for cleaved N-Term-LapA begins with Alanine 109 of the full-length protein. The arrows indicate the inferred site at which LapG cleaves LapA. (B) Alignment of putative adhesins identified by their proximity to LapG homologs. The arrow indicates the cleavage site; numbers indicate amino acid position. Organism names and an expanded alignment can be found in Figure S1. (C) Modification of WT N-Term-LapA and the cleavage site mutant (AA-RR) is compared in cell extracts with and without LapG, as visualized by Western blot. (D) A biofilm assay examining a strain carrying a chromosomal AA-RR mutation in full-length LapA, compared to that of the WT and Δ*lapG* strains. (E) LapA levels on the cell surface of the indicated strains are shown by dot blot (mean ± SD, *n* = 12). (F) LapA protein from cell extracts (Cell) and culture supernatants (Supe) is shown by Western blot.

To identify the site where N-Term-LapA is cleaved by LapG, modified and unmodified N-Term-LapA samples were purified and sequenced by Edman degradation. N-terminal sequencing revealed that the first 10 amino acids of modified N-Term-LapA are AGPSAAGTGG. These residues correspond to residues 109–118 of unmodified N-Term-LapA and chromosomally encoded LapA. Therefore, LapG functions to proteolytically cleave 108 amino acids from the N-terminus of N-Term-LapA (Figure 3A). A BlastP search with the LapG sequence helped us identify a number of LapA-like proteins encoded near LapG homologs in other bacteria. Upon aligning the N-termini of these putative adhesins, we found some residues were conserved at the site where LapA is cleaved, including alanines 108 and 109 that flank the site, as well as the position of this site relative to the N-terminus (Figures 3B and S1).

To test if conserved residues in LapA are important for recognition and/or cleavage by LapG, we constructed a mutant N-Term-LapA replacing both alanines 108 and 109 with arginine (AA-RR). Cellular extracts were prepared from WT and Δ*lapG* strains expressing WT or mutant N-Term-LapA variants and N-Term-LapA cleavage was assessed by Western blot. We observed that LapG is unable to cleave N-Term-LapA-AA-RR variant (Figure 3C), suggesting that the alanines at positions 108 and/or 109 are critical for LapG cleavage of N-Term-LapA in vitro.

The phenotype of C135A suggests that LapG-dependent cleavage of the first 108 amino acids from the N-terminus of LapA is required to release LapA from the cell surface in vivo. We therefore hypothesized that an AA-RR mutation in full-length LapA would block LapG activity in vivo and result in a hyper-adherent biofilm phenotype due to accumulation of LapA at the cell surface. We introduced the AA-RR mutation into the chromosomal copy of *lapA* by allelic replacement and assessed the biofilm phenotype. The strain expressing LapA-AA-RR forms a hyper-adherent biofilm compared to the WT, although less so than that observed for the *lapG* mutant (Figure 3D). Next we examined the effect of the AA-RR mutation on LapA accumulation. Quantitative dot blot analysis showed much higher LapA levels on the cell surface of the *lapA-*AA-RR strain compared to the WT, approaching the abundance observed for the Δ*lapG* mutant (Figure 3E). Cell extracts of these strains showed similar levels of LapA, suggesting comparable levels of LapA protein expression (Figure 3F). We saw a reduction in LapA in the culture supernatant of *lapA*-AA-RR relative to WT (down 27% ± 11% SD, *n* = 4) but not a complete loss, as observed in the Δ*lapG* mutant (Figure 3F). These results suggest that while the AA-RR mutation eliminates cleavage of N-Term-LapA in vitro, this mutation only partially blocks LapG cleavage of LapA in vivo. In support of this interpretation, introducing a *lapG* mutation into the *lapA*-AA-RR strain background yielded a hyper-adherent biofilm indistinguishable from the Δ*lapG* mutant phenotype. Importantly, these results support a model in which cleavage of the first 108 amino acids from the N-Terminus of LapA by LapG is the mechanism required to release LapA from the cell surface in vivo.

### Epistasis and Overexpression Analyses of *lapD* and *lapG*

**Figure 4:**
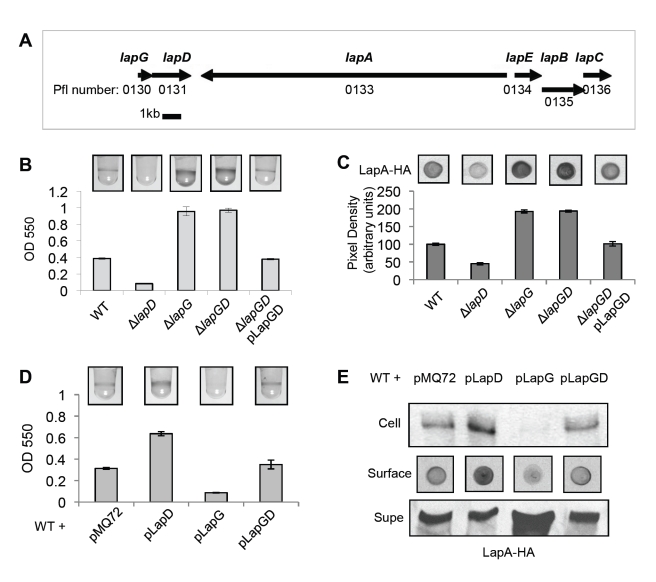
Epistasis and overexpression analyses of *lapG* and *lapD*. (A) A diagram depicting the *lap* region in the *P. fluorescens* Pf0-1 with gene names (above) and numbers (below). As shown gene sizes are approximately to scale. (B) A biofilm assay assessing the necessity of *lapG* for the *lapD* mutant phenotype. (C) The level of LapA on the surface of intact cells is quantified for strains appearing in (B) by dot blot (mean ± SD, *n* = 3). (D) A biofilm assay assessing the effects of overexpressing the indicated genes from the P_BAD_ promoter on pMQ72 by addition of 0.2% arabinose. (E) LapA levels on the cell surface are shown by dot-blot, and levels in cell extracts (Cell) and supernatant (Supe) are shown by Western blot for the same strains analyzed in (D); the growth medium contained 0.2% arabinose.

The effects of the *lapG* deletion on cell attachment and LapA localization are precisely opposite those of a *lapD* mutant (Figure 4B,C). Our previous work showed that *lapD* is required for maintenance of LapA on the cell surface; conversely, gain-of-function mutations in LapD result in biofilm and LapA localization phenotypes similar to that of a *lapG* mutant [17]. Given that LapD and LapG play opposing roles in regulating attachment via LapA, we predicted that they might function in the same pathway, and thus analyzed their genetic relationship.

The *lapG* and *lapD* genes occur in a putative operon adjacent to the genes encoding LapA and LapEBC, the ABC transporter required for LapA secretion (Figure 4A). We made a clean deletion of *lapG-lapD* and tested this strain for biofilm formation. As shown in Figure 4B and C, the *lapG lapD* double mutant (Δ*lapGD*) has a hyper-adherent biofilm phenotype and increased cell surface LapA, indicating that *lapG* is epistatic to *lapD* and that LapG likely acts downstream of LapD in controlling LapA localization. Introduction of both genes on a plasmid (pLapGD) to Δ*lapGD* was sufficient to restore WT biofilm and cell surface LapA levels (Figure 4B,C). A plasmid on which each ORF was epitope-tagged (pLapGHA-LapD6H) also complemented Δ*lapGD* (unpublished data) and was used for protein interaction studies described below.

To further explore the opposing effects of *lapG* and *lapD* on LapA localization and biofilm formation, we overexpressed each gene individually, then both simultaneously in the WT strain. Overexpressing either *lapG* or *lapD* individually phenocopied the mutant phenotype of the other gene in our biofilm assay (Figure 4D). That is, overexpression of *lapG* eliminated biofilm formation, while overexpression of *lapD* increased biofilm to levels near those of a *lapG* mutant. Importantly, expression of both genes together from the same plasmid caused no change in biofilm formation by the WT strain, indicating that the relative dosage of each protein causes the observed effects on biofilm (Figure 4D). We next examined the localization of the LapA protein in each strain. Overproduction of LapD increased LapA in the cell and at the cell surface, but decreased the amount in the supernatant (Figure 4E). Overproduction of LapG had the opposite effect: reducing cellular and cell surface LapA, while increasing the amount in the supernatant. Overexpression of both genes had no effect. Taken together, with the mutant and epistasis analyses, these data confirm that *lapG* and *lapD* exert opposing forces on the maintenance of LapA on the cell surface and suggest that they act in the same pathway.

### c-di-GMP Inhibits LapG Activity in a LapD-Dependent Manner

**Figure 5:**
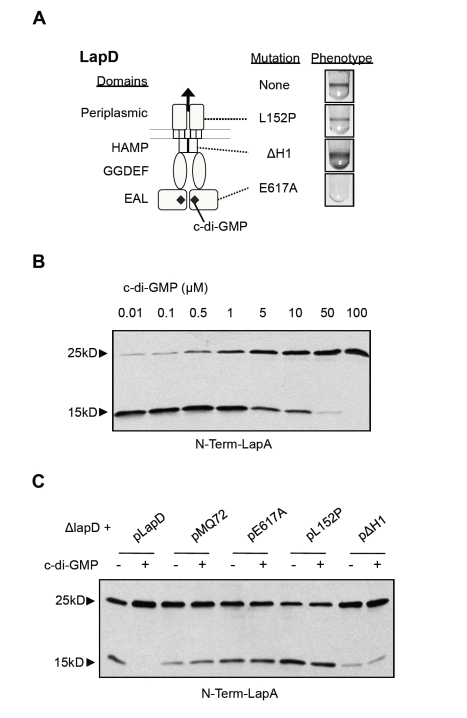
LapD-dependent effects of c-di-GMP on LapG activity. (A) A schematic diagram depicts the LapD protein as a dimer, indicating its domains (left) and biofilm phenotypes of strains carrying WT or LapD mutants (right). Dotted lines indicate the domain in which each mutation occurs. (B) N-Term-LapA (25 kD) and its cleavage product (15 kD) are visualized by anti-His Western blot, after treatment with cell extracts of the WT strain containing a range of c-di-GMP concentrations. (C) Cleavage of N-Term-LapA is visualized as in (B) after treatment with cell extracts from Δ*lapD* strains carrying the indicated plasmids, with and without 50 µM c-di-GMP.

The genetic relationship between *lapG* and *lapD* predicts a pathway in which LapD controls LapA localization through regulation of LapG's protease activity. Our previous work suggested a model whereby LapD is a transmembrane signaling protein that binds c-di-GMP via an EAL domain in the cytoplasm, and transmits this signal through a HAMP domain to a periplasmic output domain (Figure 5A) [17]. We reasoned that, when bound to c-di-GMP, LapD might inhibit LapG activity through an interaction in the periplasm.

To test this model, we first analyzed the effect of c-di-GMP on LapG's cleavage of N-Term-LapA in vitro. Addition of c-di-GMP to the lysis buffer in which cell extracts were prepared showed a dose-dependent inhibitory effect on LapG activity, consistent with first order binding kinetics (Figure 5B). Three replicate data sets were obtained for this assay and quantitative densitometry was used to determine the percentage of substrate cleaved at each concentration of c-di-GMP. A curve was fit to each data set and we estimated an average IC_50_ of LapG for c-di-GMP: 2.3±1.1 µM (Figure S2A). This value is similar to the estimated affinity of the LapD protein for c-di-GMP, a K_d_ of 5.5+2.8 µM, obtained using different methodology [17].

We next tested if inhibition of LapG activity by c-di-GMP requires LapD. Cell extracts from the *lapD* mutant carrying the empty vector pMQ72, pLapD, or pLapD mutant variants were assayed for LapG activity in the presence or absence of 50 µM c-di-GMP. Consistent with the results obtained with WT extracts, c-di-GMP addition eliminated LapG activity in extracts with functional LapD (Δ*lapD* pLapD; Figure 5C). However, in extracts that lacked LapD (Δ*lapD* pMQ72 strain), there was no effect of c-di-GMP addition on LapG activity.

To test the functional requirements for inhibition of LapG by LapD, we compared the effects of three previously characterized LapD mutants (shown in Figure 5A; [17]). A mutation in the EAL domain of LapD, E617A, shows a severe reduction in c-di-GMP binding. In a cell extract containing this LapD mutant protein, there was no inhibition of LapG by c-di-GMP (Figure 5C). The periplasmic mutation L152P reduces signaling output from LapD, and this LapD variant showed little LapG inhibition upon c-di-GMP addition (Figure 5C). Finally, the ΔH1 mutation in the HAMP domain of LapD results in constitutive signaling output regardless of c-di-GMP binding. Extracts with LapD-ΔH1 showed a severe reduction in LapG activity irrespective of c-di-GMP addition (Figure 5C). These data are fully consistent with a model in which LapD inhibits LapG activity in response to binding c-di-GMP. The effects of each LapD mutation on biofilm formation and LapA retention at the cell surface (Figure 5A) [17] are well explained by their ability to inhibit LapG.

### LapD Recruits LapG to the Inner Membrane

**Figure 6:**
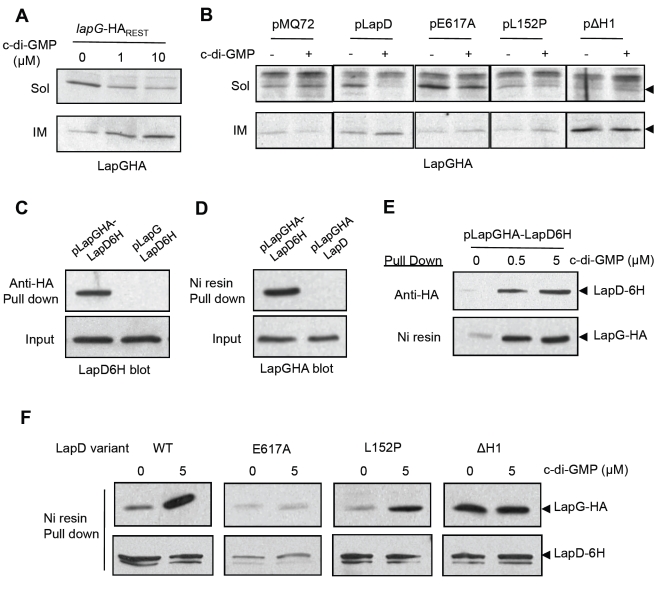
LapG interacts with LapD and is recruited to the inner membrane. (A) Subcellular fractionation was performed on the *lapG-HA_REST_* strain with the addition of c-di-GMP at the concentrations indicated. Western blots comparing levels of LapG-HA in the soluble (Sol) and inner membrane (IM) fractions are shown (additional fractionation controls can be found in Figure S3). (B) LapG-HA recruitment to the inner membrane is analyzed by Western blotting for the soluble and inner membrane fractions of the indicated strains with (+) and without (−) 10 µM c-di-GMP. (C) Immunoprecipitations with an anti-HA antibody was performed in cell extracts from the Δ*lapGD* strain expressing LapD6H and HA-tagged or untagged LapG (with 5 µM c-di-GMP). Western blots of precipitate and input are shown probed for LapD6H. (D) Co-precipitations were performed as in (C) but with a Nickel resin, using the Δ*lapGD* strain expressing LapGHA and 6H-tagged or untagged LapD. Blots were probed for LapGHA. (E) Precipitates from reciprocal pull down assays performed with cell extracts containing 0, 0.5, or 5 µM c-di-GMP are analyzed by Western blot, as indicated. (F) Nickel resin pull downs from cell extracts of strains containing pLapGHA-LapD6H with different mutations to LapD (indicated above; with and without 5 µM c-di-GMP) are shown probed by Western blot for LapGHA and LapD6H. Blot segments in (A), (B), and (F) were taken from the same or parallel blots developed with identical treatments and exposure time.

We observed that adding a number of different detergents relieved inhibition of LapG in cell extracts (Figure S2B), suggesting that membrane integrity is important for LapD to inhibit LapG. Given this observation, we hypothesized that inhibition of LapG activity by LapD is a consequence of LapD sequestering LapG to the membrane in a c-di-GMP-dependent manner. To test this idea, we looked to see if addition of c-di-GMP during cell extract preparation affected LapG localization to the inner membrane fraction. We prepared cell extracts of the *lapG-HA_REST_* strain, which carries a chromosomal copy of LapG-HA at the *lapG* locus, in buffer with 0, 1 and 10 µM c-di-GMP. Soluble and inner membrane fractions were isolated as described [24]. Addition of c-di-GMP promoted re-localization of LapG from the soluble fraction to the inner membrane fraction in a dose-dependent manner (Figure 6A), at concentrations consistent with the concentrations needed to inhibit LapG activity in cell extracts prepared under identical conditions (Figure 5B). c-di-GMP addition did not affect LapD's localization (exclusively in the inner membrane), nor did it change the localization of the cytoplasmic protein GFP (see Figure S3 for localization controls).

To determine if LapD was necessary for LapG re-localization, we disrupted the *lapD* gene in the *lapG-HA_REST_* strain. When cell fractions were prepared from the resulting strain, addition of c-di-GMP had no effect on LapG localization (Figure 6B). Interestingly, some LapG was still detected in the IM. Reintroduction of LapD on a plasmid restored c-di-GMP-dependent re-localization of LapG to the IM (Figure 6B). We also tested the functional requirements for LapD's effect on LapG by reintroducing the three LapD variants utilized above. LapD E617A is defective for c-di-GMP binding, shows no inhibition of LapG activity (Figure 5C), and nearly eliminated recruitment of LapG to the IM—even with addition of c-di-GMP (Figure 6B). The L152P mutation to LapD reduces its output [17] and LapG inhibition (Figure 5C) and also reduced LapG recruitment to the IM (Figure 6B). Lastly, the ΔH1 allele of LapD is constitutively active and strongly inhibits LapG activity; this allele promotes almost exclusive IM localization of LapG irrespective of c-di-GMP addition (Figure 6B).

### LapG and LapD Interact

To further substantiate a direct interaction between the LapG and LapD proteins, we assessed the ability of LapG and LapD to co-precipitate. First, immunoprecipitation (Ip) of HA tagged LapG was performed, and we looked for enrichment of LapD. Cell extracts were prepared from the Δ*lapGD* strain carrying pLapGHA-LapD6H, in buffer with 5 µM c-di-GMP, and 0.8% Thesit to solubilize membranes. Ip of LapGHA by the addition of anti-HA antibody and Protein A resin resulted in co-Ip of LapD6H (Figure 6C). When the assay was performed with a nearly identical strain lacking only the HA epitope on LapG, Ip of LapD6H was eliminated (Figure 6C).

We next utilized a nickel resin to pull down LapD6His and look for LapGHA co-precipitation. Precipitations were performed under the same conditions (5 µM c-di-GMP, 0.8% Thesit) with the addition of 10 mM Imidazole to reduce non-specific binding to the resin. Pull down of LapD6H enriched for LapGHA (Figure 6D). Importantly, omission of the 6H epitope from LapD eliminated precipitation of LapGHA (Figure 6D).

To examine the dependence of LapG-LapD interaction on c-di-GMP, we performed reciprocal pull down assays with 0, 0.5, or 5 µM c-di-GMP. We observed little co-precipitation in the absence of c-di-GMP but saw a dose-dependent increase when the nucleotide was added (Figure 6E). Importantly, the concentrations of c-di-GMP required to promote this interaction are similar for both types of co-precipitation. These concentrations are also on par with what is needed to recruit LapG to the inner membrane (Figure 6A), inhibit LapG activity (Figure 5B), and are consistent with the affinity of LapD for c-di-GMP.

Lastly, we introduced the E617A, L152P, and ΔH1 mutations into the pLapGHA-LapD6H plasmid to test the functional requirements for LapG-LapD interactions. The E617A LapD6H variant was not expressed at as high a level as the other alleles in this construct (Figure 6F). This is in contrast to the wild-type level of expression we have seen for this mutant from the plasmid used in our prior experiments (Figures 5C,6B; unpublished data). Despite this, we still detected some LapGHA pull down by LapD E617A, yet co-precipitation was not stimulated by c-di-GMP addition. This is consistent with the E617A mutant's defect in c-di-GMP binding (Figure 6B). LapD L152P showed a reduced ability to pull down LapGHA relative to the WT, both with and without c-di-GMP addition, consistent with reduced signaling output in this mutant (Figure 6D). Lastly, the ΔH1 mutation resulted in increased co-precipitation of LapGHA by LapD in the absence or presence of c-di-GMP, in full agreement with other data showing this allele to be constitutively active. The effects of these three mutations on LapD's ability to pull down LapG are consistent with their effects on recruitment of LapG to the inner membrane and inhibition of LapG activity. Collectively, these data describe an interaction between LapG and LapD that requires c-di-GMP binding by LapD's EAL domain, signaling through its HAMP domain and a functional periplasmic output domain.

### LapG's Role in the Phosphate-Dependent c-di-GMP Signaling Pathway

In prior publications, our group has shown that extracellular Pi is an important signal governing biofilm formation by *P. fluorescens* [17],[25]. In the absence of sufficient Pi, biofilm formation is inhibited by expression of the Pho regulon (Pho). Pho expression blocks LapA-mediated attachment in two ways: it inhibits secretion of the LapA from the cytoplasm to the cell surface, and promotes the release of the adhesin from the cell surface to the culture supernatant. Pho's effects on LapA's cell surface localization require depletion of cellular c-di-GMP by the Pho-regulated PDE RapA [25], and signaling through LapD [17]. In this study, we uncovered evidence that the output of LapD signaling is control of LapG. We next sought to test the necessity and sufficiency of the RapA-LapD-LapG signaling pathway for control of biofilm formation via LapA localization and secretion, in low Pi conditions.

**Figure 7:**
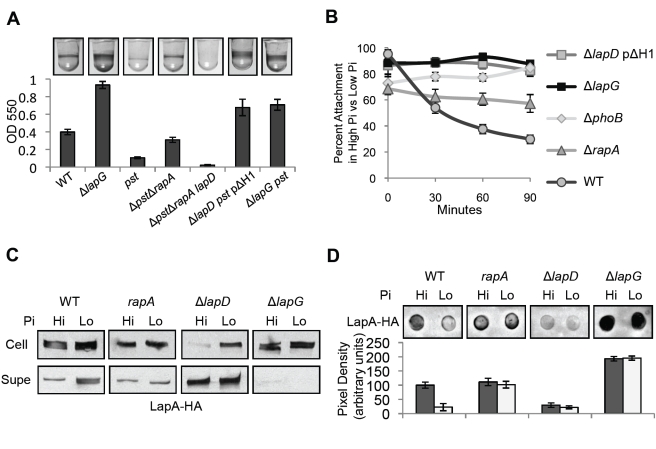
The role of the LapD-LapG system in regulation of biofilm formation by phosphate. (A) Biofilm assay documenting the impact of constitutive Pho regulon induction (via the *pst* mutation) on biofilm formation in high Pi medium (mean ± SD, *n* = 12). Constitutively active *lapD*Δ*H1* and the Δ*lapG* mutation are dominant over the *pst* mutation. (B) Physiological Pi starvation leads to detachment of biofilms from the culture well over time (see Figure S4). Here, this process is tracked by plotting the percent stained biomass attached to the well in low Pi versus high Pi medium for the indicated strains (mean ± SD, *n* = 6). (C) LapA from the whole cell fraction (Cell) into the culture supernatant (Supe) is visualized by Western blot for the indicated strains, grown in high (Hi) and low (Lo) Pi. (D) LapA on the cell surface is quantified by dot blot for samples0020analyzed in (C) (mean ± SD, *n* = 3). Blot segments in (C) and (D) were taken from the same or parallel blots developed with identical treatments and exposure time.

First, we assessed the ability of the Δ*lapG* mutation to suppress the effects of constitutive Pho regulon expression on biofilm formation. The *pst* mutant constitutively expresses Pho irrespective of Pi levels [29]; this mutation causes inhibition of biofilm formation even in high Pi medium (Figure 7A) [25]. Deletion of *rapA* in the *pst* mutant partially restores biofilm formation, to ∼70% that of the WT (Figure 7A). Pho regulon control of biofilms involves *lapD*, as the Δ*pst*Δ*rapA lapD* mutant cannot form a biofilm, and constitutively active *lapD* ΔH1 suppresses the *pst* mutation. Finally, deletion of *lapG* in the *pst* mutant leads to a hyper-adherent biofilm phenotype, despite constitutive Pho expression in this strain (Figure 7A). The level of biofilm formation by the Δ*lapG pst* strain is less than that of Δ*lapG*, but comparable to what is observed in the Δ*lapD pst* pΔH1 strain (Figure 7A). These results show that LapG plays a critical role in suppression of biofilm formation by the Pho regulon.

### LapG Is Required for Biofilm Detachment Induced by Pi Starvation

To gain insight into the dynamic response of LapD and LapG to changes in cellular c-di-GMP concentration, we evaluated the effects of physiological Pi-starvation on pre-formed biofilms. In this assay, biofilm formation proceeded identically in high Pi and low Pi media up to 5.5 h post-inoculation. At this time (designated *t* = 0) biofilms in low Pi medium began to disperse, while those in high Pi persisted at a relatively constant level for the duration of the assay (Figure 7B; biofilm images are in Figure S4). After 90 min, the WT strain showed a 70% reduction in attached biomass in low Pi, relative to the high Pi condition. The Δ*phoB* mutant showed no reduction in biofilm in low Pi, consistent with biofilm detachment requiring the activation of the Pho regulon. The *rapA* mutation partially rescues biofilm formation in low Pi [25], and here showed only 40% reduction in biofilm after 90 min in low Pi (Figure 7B). Both the Δ*lapG* and Δ*lapD* pΔH1 biofilms were unaffected by Pi starvation, showing no detachment in low Pi. These data show that Pho regulon induction leads to detachment of biofilms from the surface, and that this process requires RapA, LapD, and LapG.

### LapA Localization in Response to Pi Starvation

Pho induction inhibits secretion of LapA from the cytoplasm to the outer membrane, and also promotes its release from the cell surface into the culture supernatant [25]. To test if the RapA-LapD-LapG pathway is genetically sufficient to explain these effects, we monitored LapA localization under high and low Pi conditions in the WT, *rapA*, *lapD*, and *lapG* mutants. Consistent with our prior work, the WT strain accumulated LapA in the cellular and supernatant fractions under low Pi conditions (Figure 7C). These changes were accompanied by an 80% reduction in LapA on the cell surface when cells are grown in low Pi (Figure 7D). In contrast, the *rapA* mutant showed no apparent differences in LapA secretion between high and low Pi, and had ≈WT levels of cell surface LapA in both conditions (Figure 7C,D). These observations corroborate previous data implicating *rapA* in Pho control of both secretion and cell surface localization of LapA [25]. They suggest that c-di-GMP depletion by RapA impacts LapA in two ways: inhibiting its secretion from the cytoplasm to the outer membrane, and promoting release from the cell.

The *lapD* mutant exhibited little cell surface LapA, and abundant accumulation of LapA in the supernatant fraction irrespective of Pi concentration (Figure 7C,D). In high Pi, Δ*lapD* shows reduced LapA levels in the cellular fraction relative to WT, as reported [17]. Despite this, Δ*lapD* still accumulated intracellular LapA in low Pi (Figure 7C). This implies that, in contrast with the necessity of LapD for regulating LapA release from the cell surface, RapA controls LapA secretion in a LapD-independent manner.

**Figure 8:**
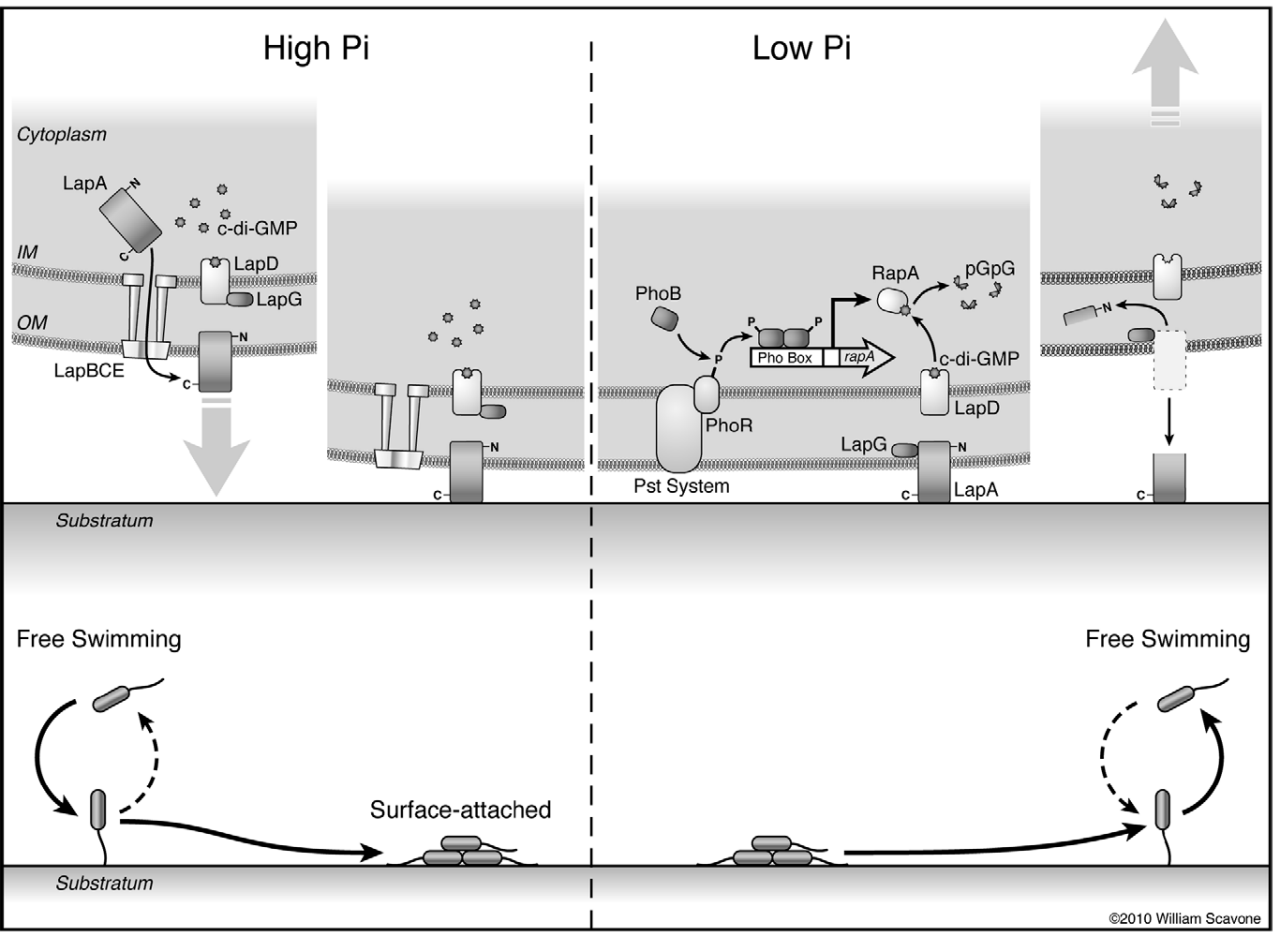
A model for regulation of biofilm formation by phosphate. This diagram depicts a summary of the current model for how Pi concentrations control biofilm formation in *P. fluorescens*. *Left*: In high Pi conditions, c-di-GMP accumulates in the cell. LapD binds c-di-GMP and sequesters LapG at the inner membrane, promoting the maintenance of LapA on the cell surface. Under these conditions, cells form irreversible attachments to the substratum and can go on to form a biofilm. *Right*: When low extracellar Pi is sensed by the PhoR/Pst system complex, the PhoR kinase is activated, and in turn phophorylates PhoB. PhoB∼P forms a dimer and binds to the Pho Box sequence upstream of *rapA*, activating its transcription. The RapA protein cleaves c-di-GMP to form pGpG, which depletes cellular c-di-GMP, leading to dissociation of c-di-GMP from LapD. Without c-di-GMP bound, LapD cannot interact with LapG, and LapG in turn cleaves the N-terminus of LapA in the periplasm, promoting its loss from the cell surface. Release of LapA from attached cells promotes their detachment from the substratum, and LapA loss from planktonic cells inhibits further surface attachment.

In low Pi, the Δ*lapG* strain showed hyper-accumulation of LapA at the cell surface, comparable to that seen in high Pi (Figure 7D). While Δ*lapG* did not release LapA into the supernatant fraction in either high or low Pi, it did show some increase in cellular LapA in low Pi (Figure 7C). Taken together, these data suggest signaling through LapD and LapG is required for release of LapA from the cell surface in response to c-di-GMP depletion by RapA (detailed in Figure 8). Our data also suggest that c-di-GMP depletion inhibits LapA secretion by a yet-unidentified LapD-independent mechanism.

## Discussion

c-di-GMP plays a key role in integrating cellular and environmental signals into a bacterium's decision to swim or stick. Recent studies highlight that c-di-GMP can impact varied outputs by binding to effector proteins, including transcription [13],[14],[15], protein localization [16], flagellar motility [9],[10],[11],[12], and EPS synthesis [6],[7],[8]. While the ubiquity and diversity of c-di-GMP signaling pathways is evident, the details of how c-di-GMP effector proteins sense and respond to their ligand are just beginning to emerge.

In *P. fluorescens* we observed release of the LapA adhesin from the cell surface in response to phosphate limitation [25]. Here we have closed a key gap in the c-di-GMP signaling pathway responsible for this effect. Together with our previous work, this study shows that LapA release depends on c-di-GMP depletion by the PDE RapA, signaling from the cytoplasm to the periplasm by the c-di-GMP effector LapD, and cleavage of the N-terminus of LapA by the protease LapG. To our knowledge, this is the most complete description of a c-di-GMP signaling “circuit” to date, linking a molecular chain of events from environmental signal to output.

Relay of a second messenger signal across the inner membrane to affect an extra-cytoplasmic output is a new paradigm in bacterial signal transduction. In the companion manuscript, Navarro et al. [26] describe structural and functional analyses of LapD, providing significant mechanistic insight into how inside-out signaling works. LapD has two stable conformations, autoinhibited and activated, and c-di-GMP binding drives conversion from the one state to the other. In the autoinhibited conformation, the “empty” EAL domain interacts with the other cytoplasmic domains, likely applying some force on the periplasmic domain and preventing it from interacting with LapG. Mutations that disrupt autoinhibtion cause hyper-adherent phenotypes in vivo, akin to that seen for the ΔH1 mutant described here (data in [26]). Our data are consistent with the ΔH1 mutation uncoupling autoinhibition from the output domain, thus causing constitutive interaction with LapG.

The L152P mutation in LapD causes reduced biofilm formation and impairs interaction with LapG, underscoring the importance of the periplasmic domain for LapD's output. How this mutation may alter LapD's conformation is not clear, as it is C-terminal to the periplasmic domain crystal structure (Navarro et al., [26]). Structure/function analyses of interactions between the purified periplasmic domain of LapD and LapG in vitro demonstrate that this domain is necessary and sufficient for LapG binding (Navarro et al., companion manuscript).

How does LapD inhibit LapG activity? One model is that LapD simply sequesters LapG at the inner membrane from its outer membrane substrate, LapA (Figure 8). It is also possible that LapD inhibits LapG enzymatic activity through allosteric or competitive means. We found no support for the latter hypothesis, observing cleavage of N-Term-LapA under in vitro conditions in which we demonstrate LapD and LapG interact (e.g. in the presence of c-di-GMP). Also, addition of excess LapD output domain had no effect on LapG activity in vitro assays with purified components (our unpublished data). These data argue for a simple sequestration model, though additional regulation cannot be ruled out.

We predict that LapG cleaves LapA in the periplasm (Figure 8). This prediction would require the N-terminus of LapA to span the outer membrane, a possibility that has yet to be investigated. LapA contains RTX motifs, which, in other proteins, can mediate interaction with and insertion into membranes [30] lending some credence to this idea. The LapG cleavage site appears to be conserved in the N-termini of other putative adhesins (Figures 3B,S1) suggesting that adhesin modification is a conserved function of LapG homologs. Additional bioinformatic analyses indicate that LapD and LapG homologs are co-conserved in putative operons, near ABC transporters and their substrates, indicating that this effector system is likely to regulate adhesin localization in many other bacteria (in [26]). A recent study on a homologous Lap system in *Pseudomonas putida* presents genetic evidence in support of this hypothesis [31].

Here we observe that activation of LapG's protease activity under low Pi conditions leads to dissolution of established *P. fluorescens* biofilms. Pho regulon induction in planktonic cells also inhibits their ability to initiate biofilms, likely due to release of LapA from the cell surface [25]. This deficiency does not impact a cell's propensity to contact the surface, however. Instead loss of LapA specifically blocks the transition from a reversible association to more stable, “irreversible” attachment [25]. Our data put c-di-GMP signaling through the LapD-LapG system at the crux of this regulatory step. The extreme phenotypes that can result from mutations to LapD, ranging from biofilm defective to hyper-adherent (Figure 5A), suggest that regulation of LapA localization by LapD-LapG sets an equilibrium between stable attachment and detachment. Loosely attached cells receiving signals that an environment is favorable may accumulate enough c-di-GMP to inhibit LapG, and initiate and maintain stable attachment via LapA. Cells that do not receive favorable signals, or firmly attached cells that sense environmental/nutritional cues that “life” is getting worse can activate LapG, allowing the cell to pick up and leave. The involvement of the LapD-LapG system in regulating both attachment to and detachment from surfaces is unique among described biofilm pathways. Whether the intrinsic reversibility of this system is common to other c-di-GMP signaling systems that regulate biofilm formation remains to be seen.

## Materials and Methods

The Supporting Information section includes additional materials and methods information (Text S1).

### Plasmid and Strain Construction

Strains and plasmids were constructed using standard molecular biology techniques and are listed in Table 1. Oligonucleotides used in this study are listed in Text S1. Detailed descriptions of strain and plasmid construction procedures can be found in the Supporting Information (Text S1).

**Table 1:** Strains and plasmids used in this study.

Strain or Plasmid	Genotype or Description	Reference
***Echerichia coli***		
Top 10	Relevant characteristics: *recA1 araD*139 Δ (ara-leu)7697	Invitrogen
S17-1(λpir)	*thi pro hsdR*− *hsdM+* Δ*recA* RP4-2::TcMu-Km::Tn7	[33]
DH5α	*supE*44 *lacU*169(80*lacZ*M15) *hsdR17 thi-1 relA1 recA1*	[34]
***Pseudomonas fluorescens***		
Pf0-1	Wild type	[35]
Δ*lapG*	Pf0-1 with unmarked deletion of *lapG*	This study
*lapG_REST_*	Δ*lapG* mutant with restored WT sequence	This study
*lapGHA_REST_*	*lapG_REST_* with an internal HA epitope	This study
Δ*lapG lapA*	Δ*lapG*::pKO-*lapA*	This study
*lapA*	Pf0-1::pKO-*lapA*	[25]
Δ*lapG lapA-HA*	Δ*lapG* expressing *lapA-HA*	This study
PF-013	Pf0-1 expressing *lapA-HA*	[25]
*lapA* AA108-109RR	PF-013 with point mutation AA108-109RR in *lapA*-HA	This study
Δ*lapD*	Pf0-1 with unmarked deletion of *lapD*	[17]
Δ*lapGD*	Pf0-1 with unmarked deletion of *lapGD*	This study
Δ*lapD lapA-HA*	Δ*lapD* expressing *lapA-HA*	[17]
Δ*lapGD lapA-HA*	Δ*lapGD* expressing *lapA-HA*	This study
*lapGHA_REST_ lapD*	*lapGHA_REST_*::pUCK-*lucK-*lapDKO	This study
*pst*	Pf0-1::pKO-*pstC*	[17]
Δ*pst*Δ*rapA*	Pf0-1 with deletions of pstSCAB-phoU and *rapA*; Gm^r^	[25]
Δ*pst*Δ*rapA lapD*	Δ*pst*Δ*rapA*::pUCK-*lucK-*lapDKO	[17]
Δ*lapD pst*	Δ*lapD*::pKO-*pstC*	[17]
Δ*lapG pst*	Δ*lapG*::pKO-*pstC*	This study
Δ*rapA*	Pf0-1 with unmarked deletion of *rapA*	[25]
*rapA lapA-HA*	PF-013::pKO-*rapA*	This study
*lapD*::*lapD6H*	Δ*lapD* with the *lapD6H* gene integrated into the native locus	[17]
**Plasmids**		
pMQ30	allelic replacement; *sacB aacC1* ColE1 oriT CEN4 URA3	[36]
pEX18-Tc	allelic replacement; *sacB tet^r^* ColE1 oriT	[37]
pEX18-LapGKO	allelic replacement construct for deletion of *lapG*	This study
pMQ30-LapGKI	allelic replacement construct for restoration of *lapG* ORF	This study
pMQ30-LapGHA-KI	allelic replacement construct for introducing *lapGHA*	This study
pKO-*lapA*	Single cross-over knockout vector for *lapA* derived from pKO3	[25]
pKO-*rapA*	Single cross-over knockout vector for *rapA* derived from pKO3	[25]
pMQ80	pMQ72 with GFP expressed from P_BAD_	[36]
pMQ71	pseudomonas expression vector; Gm^r^, Km^r^	[36]
pMini-LapA	pMQ71 expressing mini-*lapA* with the addition of myc tag	This study
pMQ72	pseudomonas expression vector; Gm^r^	[36]
pN-Term-LapA	pMQ72 expressing N-Term*-lapA*-6H	This study
pN-Term AA108-109RR	pN-Term LapA with mutation AA108-109RR in N-Term-LapA	This study
pMQ30-LapA108-109RR-KI	allelic replacement construct for introducing *lapA* AA108-109RR	This study
pLapG	pMQ72 expressing *lapG*	This study
pLapG6H	pLapG with the addition of six histidine codons to *lapG*	This study
pLapG-C135A	pLapG6H with point mutation C135A in LapG	This study
pMQ30-OperonKO	allelic replacement construct for deletion of *lapGD*	This study
pLapD	pMQ72 expressing LapD	[17]
pLapD6H	pMQ72 expressing LapD6H	[17]
pLapGHA	pMQ72 expressing LapG with internal HA epitope tag	This study
pLapGLapD	pMQ72 expressing LapGD	This study
pLapGHA LapD6H	pLapGLapD with indicated epitope tag(s)	This study
pLapGHA LapD	pLapGLapD with indicated epitope tag(s)	This study
pLapG LapD6H	pLapGLapD with indicated epitope tag(s)	This study
pLapDE617A	pMQ72 expressing LapD E617A	[17]
pLapDL152P	pMQ72 expressing LapD L152P	[17]
pLapDΔH1	pMQ72 expressing LapD ΔH1	[17]
pLapGHA LapD6HE617A	pLapGHA LapD6H with E617A mutation in LapD	This study
pLapGHA LapD6HL152P	pLapGHA LapD6H with L152P mutation in LapD	This study
pLapGHA LapD6HΔH1	pLapGHA LapD6H with ΔH1 mutation in LapD	This study
pKO-*pstC*	Single cross-over knockout vector for *pstC* derived from pKO3	[17]
pUC-*luc*K-LapDKO	*lapD* single cross-over knockout vector derived from pUC-*lucK*	[17]

### Biofilm and Surface Attachment Assays

Strains were grown statically for 6 h in K10T-1 (high Pi) medium, and biomass was stained with 0.1% crystal violet and quantified as described [25]. Data presented are means ± standard deviation (SD), *n* = 12, unless noted otherwise. For microscopy of surface attachment, strains were grown in K10T-1, and the air liquid interface imaged by phase contrast microscopy. Percent surface coverage was estimated by density measurements of digital images using ImageJ software (NIH.gov). A detailed description of the imaging and analysis procedure is in Supporting Information (Text S1). To analyze the effects of Pi starvation on biofilms, low Pi medium (K10T-π) medium was used [25].

### LapA Localization

For Western and dot blots of the LapA protein, we utilized strains with an internal 3xHA tag in chromosomal *lapA* [25]. Overnight cultures were diluted 1∶75 into K10T-1, grown for 6 h at 30°C, shaking at 230 rpm. Preparation and analysis of samples for LapA localization were performed as described previously [25]. Detection of cell surface LapA by dot blot was performed on aliquots of whole cells from the same cultures grown for LapA localization; blotting and quantification were performed as described [17]. In experiments monitoring the effects of Pi starvation on LapA localization, cultures were grown in high and low Pi media for 6.5 h.

### LapG Localization

Cultures were grown in the same manner as for LapA localization. The periplasmic fraction was obtained by incubation in osmotic shock buffer (50 mM Tris pH8, 20% sucrose, 2 mM EDTA) for 20 min at RT, followed by 10 min centrifugation at 15,000× g to pellet spheroplasts. For tracking the effect of c-di-GMP on LapG, periplasmic fractions were not prepared. Instead, clarified cell lysates were separated into soluble and membrane fractions by ultracentrifugation, 1 h at 100,000× g, and inner membranes were isolated by solubilization in 1% sarkosyl as described [24].

### Purification of Proteins

Purification of the histidine-tagged LapG, LapG-C135A, and N-Term-LapA from *E. coli* was performed using standard Nickel affinity chromatography techniques, as described [32].

### LapG Activity Assays

*Cell extract preparation*. Bacterial cultures were grown in the same manner as for LapA localization. Clarified cell extracts were prepared by sonication (4×10 s on ice) in resuspension buffer, followed by centrifugation 12 min at 15,000× g.

*Activity assays with Mini-LapA and N-Term-LapA*. To assess cleavage of Mini-LapA, cell extracts from Δ*lapG* pMini-LapA were mixed 1∶1 with cell extracts from strains with and without LapG to test their activity, and incubated at RT for 30 min. Activity assays with purified protein were performed in resuspension buffer: 750 ng N-Term-LapA (∼30 pmol; est. 95% pure) were incubated with 750 ng of LapG (est. purity: 50%) in 37.5 ul, at RT, for 15–120 min. In assays testing the site specificity of LapG, neither N-Term-LapA AA-RR nor N-term LapA were purified. Instead cleavage by endogenous LapG was assayed in cell extracts.

*Inhibition by c-di-GMP*. Chemically pure c-di-GMP (GLSynthesis Inc.) was added at various concentrations to identical aliquots of resuspended cells prior to sonication for cell extract preparation. LapG activity in cell extracts was assessed by addition of 30 pmol of purified N-Term-LapA, and incubation for 100 min at RT.

### Co-Precipitations

Proteins were precipitated from clarified lysates prepared in the same manner as for LapA localization. Immunoprecipitations, the lysis buffer contained 20 mM Tris pH 8, 10 mM MgCl_2_, and 0.8% Thesit (Sigma). The same buffer was used for nickel resin pull downs, with the addition of 10 mM imidazole. Each immunoprecipitation contained 400 µl lysate, 40 µl Protein A sepharose (Genscript), and 0.5 µl monoclonal, mouse anti-HA 11.1 antibody (Covance). Each nickel resin precipitation contained 400 µl lysate, and 40 µl resin. After incubating pull downs at 4°C for 90 min, the nickel resin (Invitrogen) was washed 2×5 min at RT with gentle shaking, then a third time briefly prior to SDS-PAGE. Pull downs with c-di-GMP added were washed with buffer containing the same concentration(s) of c-di-GMP.

### N-Terminal Sequencing

Edman degradation was performed by the Dartmouth College Proteomics Core. Details on sample preparation are included in the Supporting Information (Text S1).

## Supporting Information

Figure S1 **Expanded alignment of putative adhesins possibly cleaved by LapG homologues.** The LapG protein sequence was used as query in a BlastP search. Putative LapG homologues in related pseudomonads as well as more distantly related bacteria were identified. The genomic context of putative LapG homologues was searched visually for Type 1 secreted proteins to identify putative, LapA-like adhesins. Shown are the first 150 amino acids of 9 putative adhesins aligned with the first 150 amino acids of LapA, listed by gene name (using the AlignX program, VectorNTI suite, Invitrogen). Light grey shading indicates similar amino acids, dark grey indicates conserved amino acids, black indicates amino acids identical in all sequences. The consensus sequence is based on similar or identical residues in 7 of 10 sequences at a given position. Sequences are from the following organisms: LapA, *P. fluorescens* Pf0-1; PFL_0133, *P. fluorescens* Pf-5; PP_0168, *P. putida* KT2440; PSEEN0141, *P. entomophila* L-48; ECA3266, *Erwinia carotovora atroseptica*; DP0516, *Desulfotalea psychrophila* LSv54; Rfer_3766, *Rhodoferax ferrireducens* DSM 15236; Sden_0384, *Shewanella denitrificans* OS217; Bpro_0306, *Polaromonas* sp. JS666.
(1.62 MB TIF)

Figure S2 **Further characterization of LapG inhibition by c-di-GMP in cell extracts.** (A) Inhibition of LapG activity by c-di-GMP in cell extracts was tested over a range of concentrations, in three independent experiments. A best-fit curve was generated to estimate the apparent IC50 in each experiment, listed in the legend (right). (B) Cleavage of N-Term-LapA in WT cell extracts, with and without 50 µM c-di-GMP, is assessed by Western blot. Detergents (above) were added to the cell extracts prior to the assay at the indicated concentrations, and mixed gently for 1 min at room temperature. The first six blot segments are from one experiment, and the second two (grouped by one box) are from another. In both experiments shorter incubation times (∼40 min) were used; thus cleavage of N-Term-LapA was not complete. In most cases, detergent addition increased LapG activity relative to the no-addition control. In all cases, detergents enabled N-Term-LapA cleavage in the presence of 50 µM c-di-GMP, which completely inhibits cleavage in the absence of detergent. Detergents: LPC C-12 is lysophosphatidyl choline C-12; CHAPS is 3-[(3-Cholamidopropyl)dimethylammonio]-1-propanesulfonate; β-OG is β-octylglucoside; NP40 Alt. is nonylphenyl polyethylene glycol alternative; TX-100 is Triton X-100.
(1.05 MB TIF)

Figure S3 **Effects of c-di-GMP addition on the localization of fractionation controls.** Western blots analyzing four cellular fractions, whole cell (WC), soluble (Sol), inner membrane (IM), and outer membrane (OM), are probed for the indicated proteins. Fractionations were performed in 0, 1, or 10 µM c-di-GMP, as indicated above. Samples analyzed for LapGHA were prepared from the *lapG-HA_REST_* strain as described in the text. All other samples were prepared from a strain carrying a chromosomal copy of LapD6H and a plasmid expressing GFP (*lapD*::*lapD6H* pMQ80). While LapGHA exhibits a re-localization from the soluble to the inner membrane fraction with increasing c-di-GMP, no other protein shows this trend. The cytoplasmic protein GFP (lower band of the doublet in the WC fraction) exclusively localizes to the soluble fraction, while LapD6H exclusively localizes to the inner membrane fraction. Whole cell and membrane fractions were also probed with an antibody that recognizes OprF of *P. aeruginosa* [1], to show the relative purity of the inner and outer membrane fractions.
(1.21 MB TIF)

Figure S4 **Visualization of biofilm detachment during phosphate starvation.** Images of crystal violet stained microtiter dish biofilms, quantified (along with other replicates) for the analysis presented in Figure 7B, are shown prior to solubilization of the stain. While Figure 7B tracks the amount of biofilm stained for each strain in low Pi relative to high Pi, these images provide a visual representation of the detachment process over time. Biofilms form in both high and low Pi medium and are roughly equivalent at 5.5 h post-inoculation. After 5.5 h, the WT biofilm gradually detaches from the well in low Pi medium. Detachment requires Pho regulon expression (there is no detachment in the Δ*phoB* mutant) and is largely explained by expression of the Pho-regulated c-di-GMP PDE RapA (there is reduced detachment in the *rapA* mutant). A constitutively active allele of LapD (ΔH1) and the Δ*lapG* mutation both confer complete insensitivity to Pi starvation.
(1.40 MB TIF)

Text S1 Detailed materials and methods.
(0.10 MB DOC)

## Abbreviations

BTLCP: Bacterial Transglutaminase-like Cysteine Proteinase
c-di-GMP: cyclic dimeric GMP
DGCs: diguanylate cyclases
EPS: exopolysaccharide
PDE: phosphodiesterase
Pi: inorganic phosphate
