# Supplementary figures and images for: A c-di-GMP Effector System Controls Cell Adhesion by Inside-Out Signaling and Surface Protein Cleavage
Source: PLoS Biol. 2011 Feb 1;9(2):e1000587. doi: 10.1371/journal.pbio.1000587 (PMC3032545; doi:10.1371/journal.pbio.1000587)

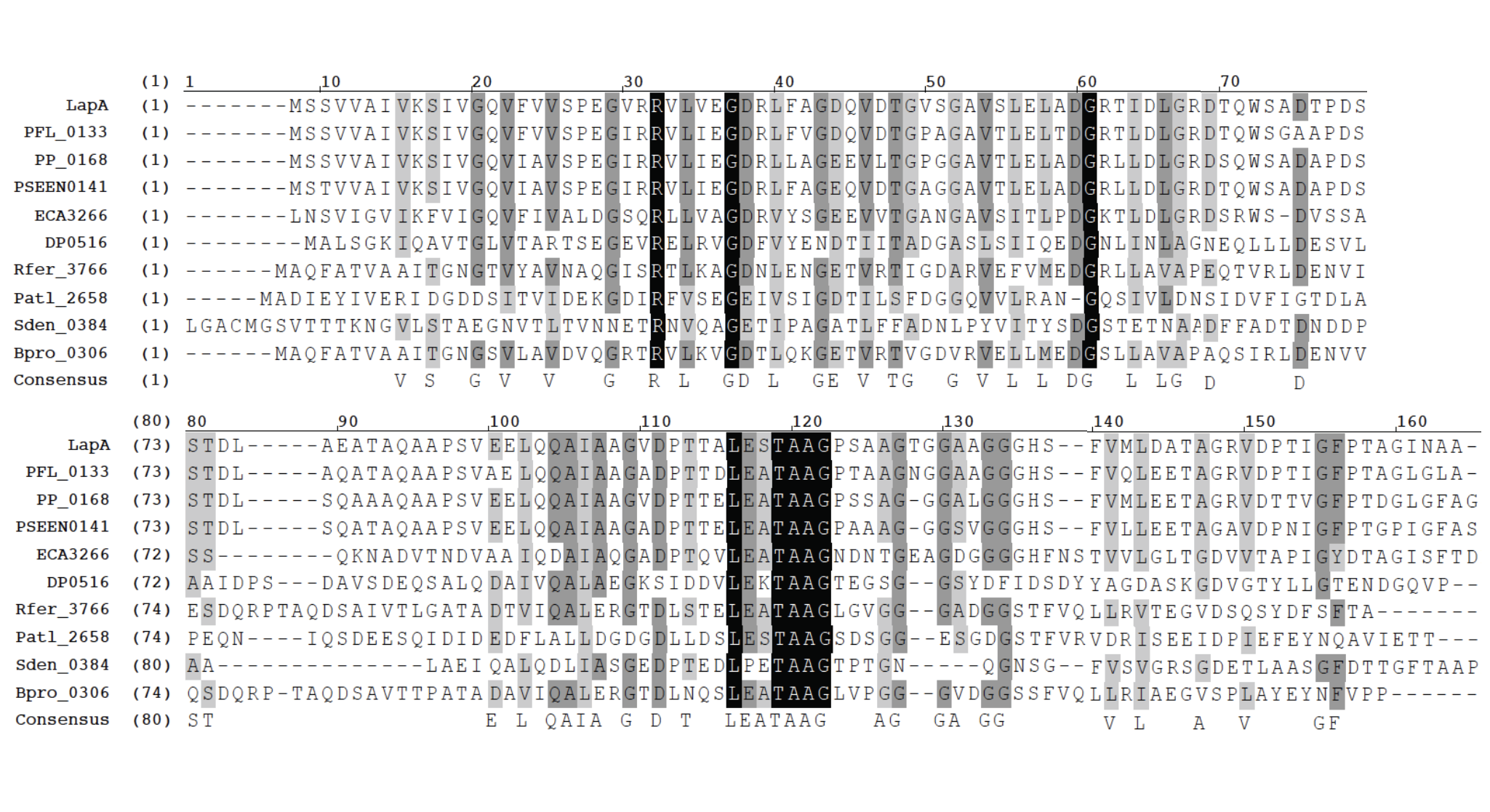

Supplement: Figure S1 — Expanded alignment of putative adhesins possibly cleaved by LapG homologues. The LapG protein sequence was used as query in a BlastP search. Putative LapG homologues in related pseudomonads as well as more distantly related bacteria were identified. The genomic context of putative LapG homologues was searched visually for Type 1 secreted proteins to identify putative, LapA-like adhesins. Shown are the first 150 amino acids of 9 putative adhesins aligned with the first 150 amino acids of LapA, listed by gene name (using the AlignX program, VectorNTI suite, Invitrogen). Light grey shading indicates similar amino acids, dark grey indicates conserved amino acids, black indicates amino acids identical in all sequences. The consensus sequence is based on similar or identical residues in 7 of 10 sequences at a given position. Sequences are from the following organisms: LapA, P. fluorescens Pf0-1; PFL_0133, P. fluorescens Pf-5; PP_0168, P. putida KT2440; PSEEN0141, P. entomophila L-48; ECA3266, Erwinia carotovora atroseptica; DP0516, Desulfotalea psychrophila LSv54; Rfer_3766, Rhodoferax ferrireducens DSM 15236; Sden_0384, Shewanella denitrificans OS217; Bpro_0306, Polaromonas sp. JS666. (1.62 MB TIF) [file pbio.1000587.s001.tif]

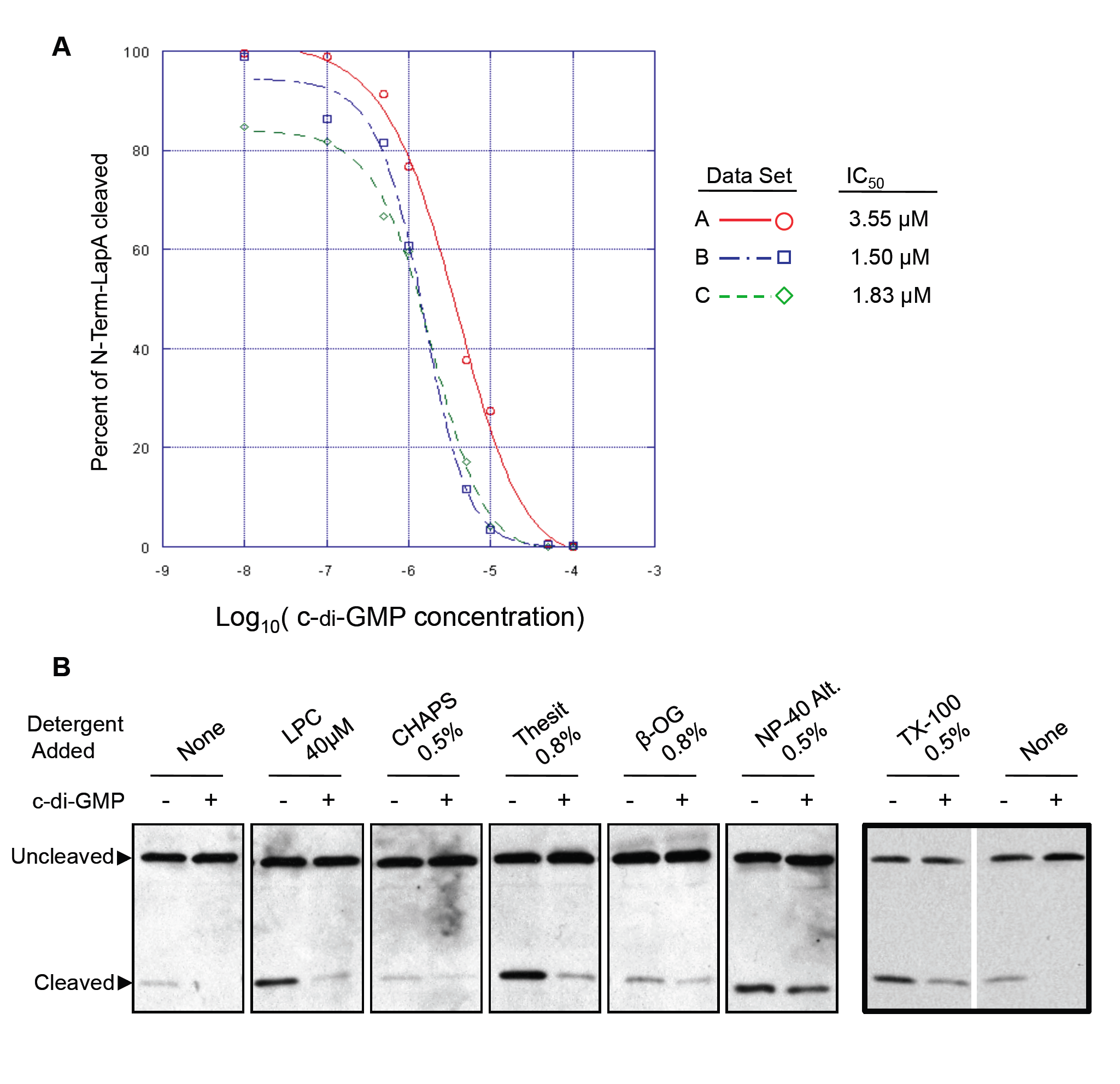

Supplement: Figure S2 — Further characterization of LapG inhibition by c-di-GMP in cell extracts. (A) Inhibition of LapG activity by c-di-GMP in cell extracts was tested over a range of concentrations, in three independent experiments. A best-fit curve was generated to estimate the apparent IC50 in each experiment, listed in the legend (right). (B) Cleavage of N-Term-LapA in WT cell extracts, with and without 50 µM c-di-GMP, is assessed by Western blot. Detergents (above) were added to the cell extracts prior to the assay at the indicated concentrations, and mixed gently for 1 min at room temperature. The first six blot segments are from one experiment, and the second two (grouped by one box) are from another. In both experiments shorter incubation times (∼40 min) were used; thus cleavage of N-Term-LapA was not complete. In most cases, detergent addition increased LapG activity relative to the no-addition control. In all cases, detergents enabled N-Term-LapA cleavage in the presence of 50 µM c-di-GMP, which completely inhibits cleavage in the absence of detergent. Detergents: LPC C-12 is lysophosphatidyl choline C-12; CHAPS is 3-[(3-Cholamidopropyl)dimethylammonio]-1-propanesulfonate; β-OG is β-octylglucoside; NP40 Alt. is nonylphenyl polyethylene glycol alternative; TX-100 is Triton X-100. (1.05 MB TIF) [file pbio.1000587.s002.tif]

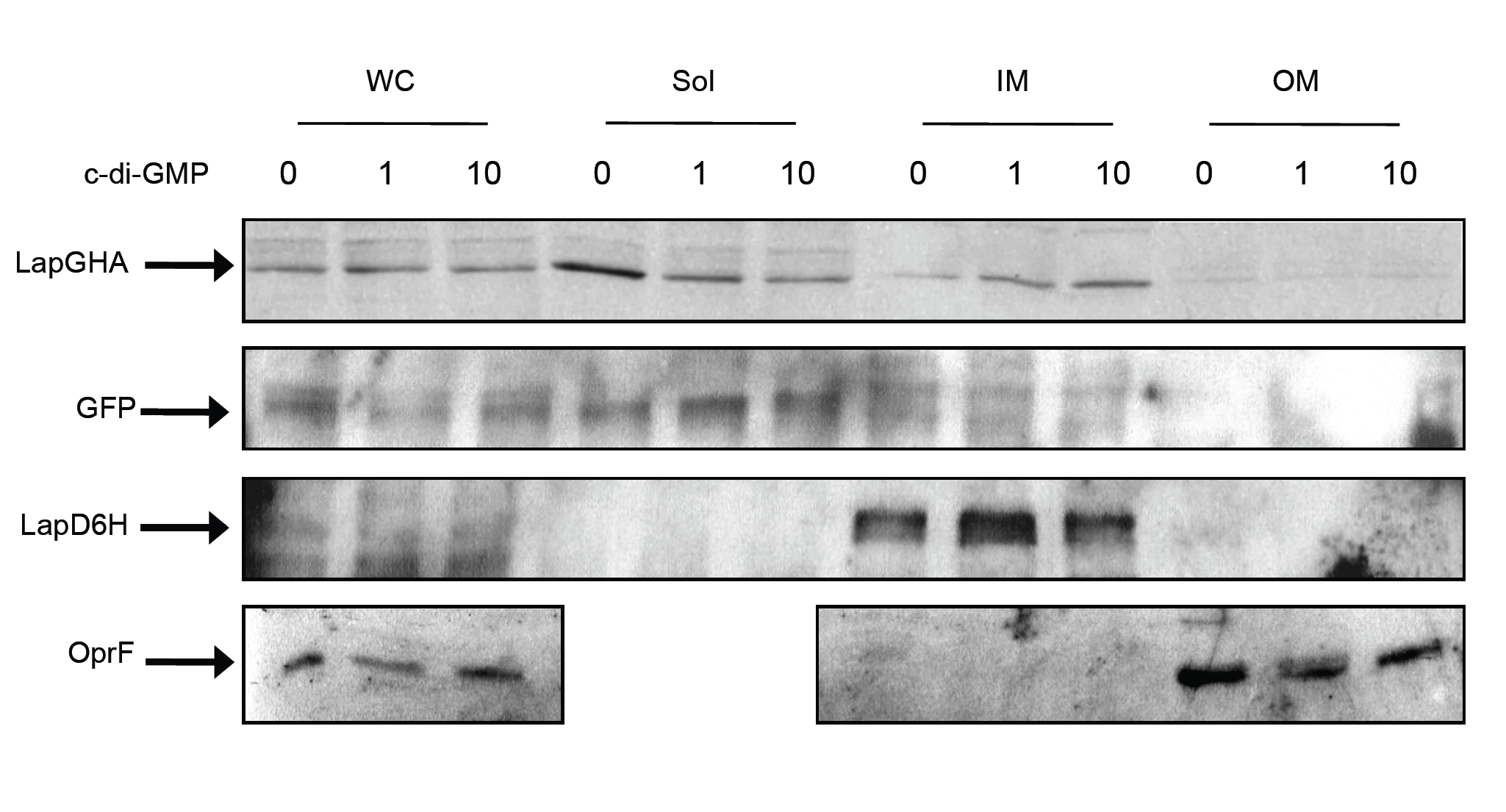

Supplement: Figure S3 — Effects of c-di-GMP addition on the localization of fractionation controls. Western blots analyzing four cellular fractions, whole cell (WC), soluble (Sol), inner membrane (IM), and outer membrane (OM), are probed for the indicated proteins. Fractionations were performed in 0, 1, or 10 µM c-di-GMP, as indicated above. Samples analyzed for LapGHA were prepared from the lapG-HAREST strain as described in the text. All other samples were prepared from a strain carrying a chromosomal copy of LapD6H and a plasmid expressing GFP (lapD::lapD6H pMQ80). While LapGHA exhibits a re-localization from the soluble to the inner membrane fraction with increasing c-di-GMP, no other protein shows this trend. The cytoplasmic protein GFP (lower band of the doublet in the WC fraction) exclusively localizes to the soluble fraction, while LapD6H exclusively localizes to the inner membrane fraction. Whole cell and membrane fractions were also probed with an antibody that recognizes OprF of P. aeruginosa [1], to show the relative purity of the inner and outer membrane fractions. (1.21 MB TIF) [file pbio.1000587.s003.tif]

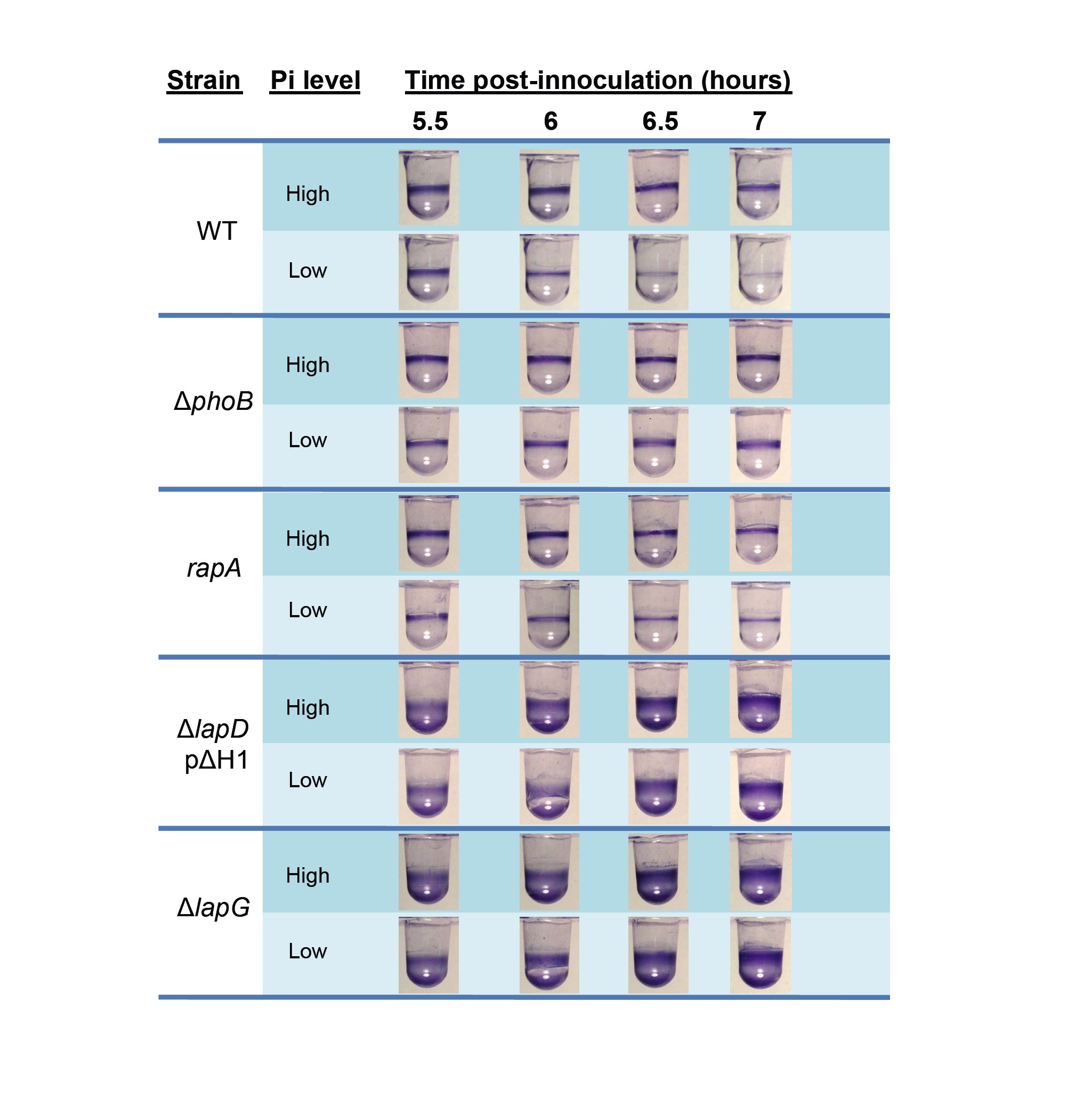

Supplement: Figure S4 — Visualization of biofilm detachment during phosphate starvation. Images of crystal violet stained microtiter dish biofilms, quantified (along with other replicates) for the analysis presented in Figure 7B, are shown prior to solubilization of the stain. While Figure 7B tracks the amount of biofilm stained for each strain in low Pi relative to high Pi, these images provide a visual representation of the detachment process over time. Biofilms form in both high and low Pi medium and are roughly equivalent at 5.5 h post-inoculation. After 5.5 h, the WT biofilm gradually detaches from the well in low Pi medium. Detachment requires Pho regulon expression (there is no detachment in the ΔphoB mutant) and is largely explained by expression of the Pho-regulated c-di-GMP PDE RapA (there is reduced detachment in the rapA mutant). A constitutively active allele of LapD (ΔH1) and the ΔlapG mutation both confer complete insensitivity to Pi starvation. (1.40 MB TIF) [file pbio.1000587.s004.tif]
