# Supplementary material for: A c-di-GMP Effector System Controls Cell Adhesion by Inside-Out Signaling and Surface Protein Cleavage
Source: PLoS Biol. 2011 Feb 1;9(2):e1000587. doi: 10.1371/journal.pbio.1000587 (PMC3032545; doi:10.1371/journal.pbio.1000587)
Supplement: Text S1 — Detailed materials and methods. (0.10 MB DOC) [file pbio.1000587.s005.doc]

**Detailed Materials and Methods**

**Construction of plasmids and strains**

Microbiological methods:

For routine culture, *P. fluorescens* and *E. coli* were grown in lysogeny broth (LB) at 30°C and 37°C, respectively. When appropriate, antibiotics were added to the medium at the following concentrations: *E. coli-* 10μg/ml Tetracycline, 10μg/ml Gentamycin; *P. fluorescens-* 15μg/ml Tetracycline, 30μg/ml Gentamycin. Replicative plasmids were introduced to *E. coli* and *P. fluorescens* by electroporation by standard techniques. Non-replicating plasmids were introduced into *P. fluorescens* by conjugation. The yeast strain InvSc1 (*Saccharomyces cerevisiae*; Invitrogen), was routinely cultured on YPD medium. When selecting for plasmids carrying the *URA3* gene it was grown on YNB with complete supplemental mixture minus uracil.

Yeast cloning for plasmid construction: All oligonucleotide primers used in the study are listed in table S1. Unless otherwise noted, cloning was performed by in vivo recombination in yeast, as described [2]. Briefly, vectors bearing the *2μ* replication origin and *URA3* gene were linearized by restriction enzyme digestion. One or more inserts were generated by PCR using primers with 30 or more non-annealing bases to facilitate homologous recombination with adjacent fragments and/or the vector. Then, PCR fragments and linearized vector were introduced into yeast by transformation and recombinant vector bearing insert(s) of interest was selected for on uracil minus medium. All PCR was performed using Phusion polymerase (Finzymes).

Allelic replacement: Plasmid and strain construction

Plasmids based on the pMQ30 allelic replacement vector, were used to generate the following knockout and knock-in strains in WT and *lapA-HA* backgrounds: Δ*lapGD*, *lapG_REST_*, *lapGHA_REST_*, *lapA* AA108-109RR. The same basic technique was employed in all cases. Two stretches of homologous DNA flanking the genomic region to be deleted or replaced were amplified by PCR (700-1000bp each), utilizing primers with 30 or more extra bases to facilitate recombination with adjacent fragments in cloning. For knock-in mutations, the replacement alleles were included between the flanking fragments such that recombination with the chromosome would reconstitute the native locus with only the desired change from WT; in deletion constructs nothing was cloned between flanking fragments. Allelic replacement was performed as previously described [3]. Briefly, constructs were introduced into *P. fluorescens* by conjugation with *E. coli*, and transconjugates selected for on LB 30μg/ml Gentamycin and 30μg/ml Chloramphenicol. After verifying single-crossover insertion of constructs in the chromosome by PCR, strains were cultured O/N without antibiotic, then plated on LB with 0.5% (wt/vol) sucrose to select for the second crossover event. Mutants were verified by PCR and sequencing of purified genomic DNA to ensure proper construction.

Construction of pEX18-LapGKO

Allelic replacement vector pEX18-Tc was used for making the Δ*lapG* strain. Upstream and downstream sequences flanking the *lapG* reading frame were amplified individually, using primers including restriction enzyme sites, oriented as follows: HindIII-Upstream-XhoI, XhoI-Downstream-EcoRI. PCR fragments were cut with XhoI, ligated with T4DNA ligase (Invitrogen), then the product of this reaction used as template for a second PCR reaction using Upstream foward and Downstream reverse primers. The product, containing both Upstream and Downstream sequences in the same fragment was cut with HindIII and EcoRI, then ligated to pEX18-Tc that had been cut with the same enzymes. The resulting construct, pEX18-LapGKO, was employed in the same fashion as pMQ30-based constructs described above to generate an unmarked deletion of the *lapG* gene. All restriction enzymes were obtained from New England Biolabs.

Construction and modification of expression plasmids

All expression constructs were built in pMQ72 downstream of the P_BAD_ promoter, with the exception of pMini-LapA which was built in a nearly identical plasmid (pMQ71) which carries an additional, unused, antibiotic marker (*kanR*). These include: pN-Term-LapA, pLapGD and derivatives. First, native sequences were amplified by PCR from genomic DNA using primers with additional non-annealing bases to facilitate recombination with the vector during yeast cloning. For the addition of histidine tags, additional codons (CAT) were incorporated into the primers in the desired position relative to the native ORF. Most site-directed mutations and internal epitope tags were added to expression constructs using the same technique. First, the ORF to be modified was amplified in two pieces, the 5’ fragment ending just upstream of the codon or site to be changed and the 3’ fragment beginning just downstream. The new codon (or additional codons for epitope tags) and 21 complimentary bases were added to each internal primer such that recombination in yeast would yield the desired change in the translated ORF. When possible, sequenced plasmids containing the ORFs to be modified were used as template. All variants were confirmed by DNA sequencing.

Construction of pLapG6H

The expression plasmid for LapG was cloned by incorporating an EcoRI site into the forward primer and HindIII site into the reverse primer. The PCR product was cut with these restriction enzymes, then ligated to EcoRI/HindIII-cut pMQ72.

Constructing Variants of pLapGD

Variants of pLapGD carrying epitope tags and point mutations were constructed as follows. LapG and LapD ORFs were amplified independently utilizing a reverse and forward primer (respectively) that overlap (LapG Rev, Intergenic Fwd), and external primers that recombine with the vector. Differentially epitope tagged variants as well as variants with mutations to LapD were constructed with the same primer sets, utilizing templates carrying the desired mutation(s).

Internal Epitope tagging of LapG

When building an epitope tagged LapG we were sensitive to potential effects on interaction with or expression of LapD. Thus, four different placements for the tag were tested in parallel, and built in pLapGLapD6H for easy assessment of function and expression of both genes. Only LapG variants that were active and still interacting with LapD would yield a WT phenotype when this plasmid is introduced into a Δ*lapGD* strain. Of four variants, only one yielded a WT phenotype. This plasmid was built by amplifying one fragment consisting of the N-terminal 219 codons of LapG with the addition of codons for one HA epitope flanked by linker amino acids, and a second fragment beginning at codon 218 of LapG and extending through LapD6H on pLapGLapD6H. Recombination of these fragments with pMQ72 in yeast yielded pLapGHALapD6H in which 16 additional codons encoding QNLLYPYDVPDYAAGA occur between codons 218 and 219 in *lapG.*

**Estimation of surface coverage in microscopy images** (Related to Figure 1B)

For phase contrast microscopy studies of surface attachment, strains were grown statically in 1ml K10T-1 in a 12-well polystyrene cell culture dish for 1 hour. The dish was elevated at one end to position the air liquid interface (ALI) in the center of the bottom of each well. Prior to imaging, wells were washed twice with fresh medium to remove reversibly attached cells, and then 1ml of fresh medium was added. Phase contrast microscopy with a Zeiss Axiovert 200M, and 63x long-working-distance objective was used to visualize the band of attached cells below the ALI (several hundred μm wide). Images of eight fields of view were captured for each strain, all within 50 μm of the ALI, using the same light and camera settings. To estimate surface coverage by the cells, an integrated pixel density measurement was taken of each image, in which cells appear as dark rods against a light background (Figure 1B), using ImageJ software (http://rsbweb.nih.gov/ij/). These raw values were normalized to the background density in an image of an uncolonized surface, and compared relative to the pixel density of a confluent monolayer of attached cells (set as 100% coverage) taken at the 4 hour time point of the same assay.

**Sample preparation for N-terminal sequencing** (Related to Figure 3A)

To prepare samples for N-terminal sequencing, a 400ul volume activity assay containing 15mg/ml of purified N-Term-LapA-His and 30mg/ml of a cellular extract prepared from Δ*lapG* pLapG was carried out at room temperature for 1 hour. HisLink protein purification resin (Promega) was equilibrated with binding buffer (20mM Na_2_PO_4_, pH 8, 0.5M NaCl, 20mM imidazole). The activity assay was added to 400ul of the His resin and incubated for 2 hours with constant gentle shaking. Following a 5-minute centrifugation at 10,000 rpm, the resin was washed twice with 100% binding buffer and subsequently centrifuged. To elute background, the resin was washed with 75% binding buffer and 25% elution buffer (20mM Na_2_PO_4_, pH 8, 0.5M NaCl, 200mM imidazole), centrifuged, and washed again with 45% binding buffer and 55% elution buffer. To elute cleaved N-Term-LapA the resin was washed with 100% elution buffer and then centrifuged. Cleaved N-Term-LapA was concentrated with a Millipore Amicon Ultra-0.5 Centrifugal Filter Unit MW 10kDa (Fisher Scientific), to ~100ul and approximately 0.9mg/ml. All steps were performed at 4°C unless otherwise noted. 37.5ul of the concentrated cleaved N-Term-LapA and 37.5ul of 1mg/ml of uncleaved N-Term-LapA were analyzed by SDS-PAGE and transferred to a PVDF membrane. Following coomassie staining of the membrane, both cleaved and uncleaved N-Term-LapA samples were cut from the membrane and then analyzed by Edman degradation at the Dartmouth College Molecular Biology and Proteomics Core.

**Procedure for IC_50_ estimation** (Related to Figure 5B and S1)

To estimate the IC_50_ of LapG for c-di-GMP, we tracked cleavage of N-Term-LapA in cell extracts by western blot. First, identical aliquots of cells were lysed by sonication in resuspension buffer amended with the following concentrations of c-di-GMP: 0.01, 0.05, 0.1, 0.5, 1, 5, 10, 50 or 100μM. Clarified cell extracts were prepared from lysates as described above, and normalized to a total protein concentration of 4mg/ml. For each cleavage assay, 2μl of cell extract were mixed with 30 pmol of purified substrate in a volume of 30μl, and incubated at RT for 100 min prior to western blot analysis.

Western blots were scanned with HP Scanjet® and converted to TIFF files. The percentage of substrate cleaved was determined by making integrated density measurements using ImageJ software (http://http://rsbweb.nih.gov/ij/): (Total density of both cleaved and uncleaved N-Term-LapA) minus (density of cleaved band) times 100. Percentage values for three independent experiments were plotted against the Log of the concentration c-di-GMP using KaleidaGraph (Synergy Software). Best fit curves and IC_50_ estimates were generated as per the manufacturer’s instructions (Fig. S2).

**Supporting Information References**

1. MacEachran DP, Stanton BA, O'Toole GA (2008) Cif is negatively regulated by the TetR family repressor CifR. Infect Immun 76: 3197-3206.

2. Shanks RM, Caiazza NC, Hinsa SM, Toutain CM, O'Toole GA (2006) Saccharomyces cerevisiae-based molecular tool kit for manipulation of genes from gram-negative bacteria. Appl Environ Microbiol 72: 5027-5036.

3. Monds RD, Newell PD, Schwartzman JA, O'Toole GA (2006) Conservation of the Pho regulon in Pseudomonas fluorescens Pf0-1. Appl Environ Microbiol 72: 1910-1924.

| **Table S1.** Oligonucleotide primers used in this study^a^ | | |  |
| --- | --- | --- | --- |
| **No.** | **Sequence** | | **Information** |
| 1 | 5'- | ATACCCGTTTTTTTGGGCTAGCGAATTCGAGCTCGGTACCCCGGAGAGTCTTCAATGAGCAGTG | N-Term-LapA Fwd |
| 2 | 5'- | tcttctGGTGGATTCAAGGGCGGTAGTC | Internal Rev AA-RR mutation |
| 3 | 5'- | GACTACCGCCCTTGAATCCACCagaagaGGCCCGAGCGCCGCTG | Internal Fwd AA-RR mutation |
| 4 | 5'- | CTCTAGAGGATCCCCTTAATGATGATGATGATGATGTCCAACAGTGTCGTTCGGTGCCAG | N-Term-LapA Rev |
| 5 | 5'- | CCGTTTTTTTGGGCTAGCGAATTCGAGCTCGGTACCCCCAGCGATGATCTCAGGGTGCC | LapG ORF Fwd for pMQ72 |
| 6 | 5'- | ACTTGGCGATCGCGTAGTCTTCTGCGTCGCCGGCGCCTTTCCACAAG | Internal Rev C135A mutation |
| 7 | 5'- | GCAGAAGACTACGCGATCGCCAAG | Internal Fwd C135A mutation |
| 8 | 5'- | CCGCCAAAACAGCCAAGCTTG | LapG ORF Rev |
| 9 | 5'- | cagctatgaccatgattacgaattcgagctcggtaccc gctggatctggtcgtagatgcgc | Upstream flank for *lapA* AA-RR-KI Fwd |
| 10 | 5'- | aagcttgcatgcctgcaggtcgactctagaggatccccGTCGCTGGCGTCGTGTTCG | Downstream flank for *lapA* AA-RR-KI Rev |
| 11 | 5'- | atacccgtttttttgggctagcgaattcgagctcggtacccCGGAGAGTCTTCAATGAGCAGTG | Mini-LapA N-terminus fwd |
| 12 | 5'- | cacgcctaaatcttcttcagaaattaatttttgttcAACAGTGTCGTTCGGTGCCAG | Mini-LapA N-terminus rev |
| 13 | 5'- | actgttgaacaaaaattaatttctgaagaagatttaGGCGTGTACACCGTGAAGATCC | Mini-LapA C-terminus fwd |
| 14 | 5'- | gccaagcttgcatgcctgcaggtcgactctagaggatccccCCAACTGCGCACGAGTATGC | Mini-LapA Cterminus rev |
| 15 | 5'- | attgaattcCCAGCGATGATCTCAGGGTG | LapG6H fwd (EcoRI site) |
| 16 | 5'- | tttaagcttTTAATGATGATGATGATGATGCGCGTGCTCCTTGTTAGTCGTCTCG | LapG6H rev (HindIII site) |
| 17 | 5'- | atttaagcttGATTGAGGTGCTGCTCCAG | LapGKO Upstream fwd (HindIII site) |
| 18 | 5'- | gaattctagaGCTTGATGGTGAAGGTTAGCC | LapGKO Upstream rev (XhoI site) |
| 19 | 5'- | cttatctagaGCTGGCAGGATGTGTTGAAG | LapGKO Downstream fwd (XhoI site) |
| 20 | 5'- | ctaagaattcGGTAGGACTCGGTACGCAG | LapGKO Downstream rev (EcoRI site) |
| 21 | 5'- | ATGACCATGATTACGAATTCGAGCTCGGTACCCGATTGAGGTGCTGCTCCAGCAG | LapGKI fwd |
| 22 | 5'- | GccaagcttgcatgcctgcaggtcgactctagaggatccccGGCACTGTCGAGACTCTGCG | LapGKI rev |
| 23 | 5'- | GCTTGATGGTGAAGGTTAGCCCAG | LapGHA-KI Upstream flank rev |
| 24 | 5'- | TCATCATCATTAAGGGGCTGACTGATCTTTCCCACGCTCC | LapGHA-KI Downstream flank fwd |
| 25 | 5'- | GccaagcttgcatgcctgcaggtcgactctagaggatccccGGCAACGACACGCTGATCGG | LapGHA-KI Downstream flank rev |
| 26 | 5'- | GCGATGATCTCAGGGTGCCATTG | LapGHA ORF for KI 5’ fwd |
| 27 | 5'- | GccaagcttgcatgcctgcaggtcgactctagaggatccccGGCACTGTCGAGACTCTGCG | LapGHA ORF for KI 3’ rev |
| 28 | 5'- | CAGCTATGACCATGATTACGAATTCGAGCTCGGTACCCGATTGAGGTGCTGCTCCAGC | OperonKO Upstream Fwd |
| 29 | 5'- | GGA GCG TGG GAA AGA TCA GTCAGCGCTTGATGGTGAAGGTTAGCC | OperonKO Upstream Rev |
| 30 | 5'- | GGC TAA CCT TCA CCA TCA AGCGCTGACTGATCTTTCCCACGCTC | OperonKO Downstream Fwd |
| 31 | 5'- | CaagcttgcatgcctgcaggtcgactctagaggatccGGCAACGACACGCTGATCG | OperonKO Downstream Rev |
| 32 | 5'- | TTTTTTTGGGCTAGCGAATTCGAGCTCGGTACCCCAATGGCACCCTGAGATCATCGC | LapG for pMQ72 Fwd |
| 33 | 5'- | AAAGACATCTGAGCGCGTGCTCCTAGTTAGTCGTCTCGAC | LapG ORF for pMQ72 Rev |
| 34 | 5'- | GTCGAGACGACTAACTAGGAGCAC | Intergenic (upstream of LapD) Fwd |
| 35 | 5'- | CAGGCTGAAAATCTTCTCTCATCCG | pMQ72 Rev for variations on pLapGD |
| 36 | 5'- | gtagagcaggttctgCGCTCCCGGCAGGTACAAACC | Internal for LapGHA tag rev |
| 37 | 5'- | atatgacgttccagattacgctgctGGAGCGAAGGGCAACAAGAAGG | Internal for LapGHA tag fwd |
| 38 | 5'- | cttgcatgcctgcaggtcgactctagaggatccccGAGCGTGGGAAAGATCAGTCAG | LapD (no tag) for pLapGD rev |

^a^ Underined sequences denote non-annealing bases employed for recombination in yeast or for the
 addition of restriction enzyme sites and/or epitope tags.
